# Luteolin regulates the distribution and function of organelles by controlling SIRT1 activity during postovulatory oocyte aging

**DOI:** 10.3389/fnut.2023.1192758

**Published:** 2023-07-31

**Authors:** Xupeng Xing, Jingfeng Peng, Jingyu Zhao, Ruoxi Shi, Caiqin Wang, Zihan Zhang, Zihan Wang, Zicong Li, Zhenfang Wu

**Affiliations:** ^1^National Engineering Research Center for Breeding Swine Industry, South China Agricultural University, Guangzhou, China; ^2^Department of Animal Genetics, Breeding and Reproduction, College of Animal Science, South China Agricultural University, Guangzhou, China; ^3^Collaborative Innovation Center for Birth Defect Research and Transformation of Shandong Province, Jining Medical University, Jining, China; ^4^College of Second Clinical Medical, Jining Medical University, Jining, China

**Keywords:** luteolin, postovulatory oocyte aging, aging-induced changes, organelles, SIRT1

## Abstract

The quality of oocytes determines their development competence, which will be rapidly lost if the oocytes are not fertilized at the proper time after ovulation. SIRT1, one of the sirtuin family members, has been proven to protect the quality of oocytes during postovulatory oocyte aging. However, evidence of the effect of SIRT1 on the activity of organelles including the mitochondria, the endoplasmic reticulum (ER), the Golgi apparatus, and the lysosomes in postovulatory aging oocyte is lacking. In this study, we investigated the distribution and function of organelles in postovulatory aged oocytes and discovered abnormalities. Luteolin, which is a natural flavonoid contained in vegetables and fruits, is an activator of SIRT1. When the oocytes were treated with luteolin, the abnormal distribution of mitochondria, ER, and Golgi complex were restored during postovulatory oocyte aging. The ER stress protein GRP78 and the lysosome protein LAMP1 increased, while the mitochondrial membrane potential and the Golgi complex protein GOLPH3 decreased in aged oocytes, and these were restored by luteolin treatment. EX-527, an inhibitor of SIRT1, disrupted the luteolin-mediated normal distribution and function of mitochondria, ER, Golgi apparatus, and lysosomes. In conclusion, we demonstrate that luteolin regulates the distribution and function of mitochondria, ER, Golgi apparatus, and lysosomes during postovulatory oocyte aging by activating SIRT1.

## Introduction

From 15–20% of couples in developed countries and China, face problems of infertility and sterility necessitating dependence on artificial assisted reproduction (1). Oocyte quality is critical for their development after fertilization and poor quality severely limits successful reproductive outcomes (2). After ovulation, the matured oocytes are arrested at the metaphase II (MII) stage and the MII oocytes do not continue into the next stage of development if they are not be fertilized (3). The quality of MII oocytes rapidly decreases if they not fertilized at proper time, and this is referred to as postovulatory oocyte aging. In artificial assisted reproduction in humans, if the oocytes failed to respond to *in vitro* fertilization (IVF), the rescued intracytoplasmic sperm injection could be subsequently applied (4). Therefore, postovulatory oocyte aging is unavoidable during artificial assisted reproduction. The aging oocyte lacks developmental competence resulting in a low rate of fertilization, poor blastocyst formation, low pregnancy rate and deficiency in healthy offspring (5–7). In an effort to mitigate this serious problem, we chose to focus on potential regulatory mechanisms of postovulatory aging.

Normally functioning organelles including mitochondria, the ER, the Golgi apparatus, and lysosomes are critical for oocyte maturation and embryo development. Mitochondria are responsible for energy production, calcium homeostasis, cytoplasmic redox regulation, and signal transduction (8). Because mitochondria are maternally inherited and their replication does not begin until implantation, healthy mitochondria are critically important for the development of competent oocytes and embryos (8, 9). The distribution and function of mitochondria was shown to be abnormal in aged oocytes from mice, and this could be corrected by antioxidants treatment (5, 10). The ER is responsible for the synthesis of properly folded proteins for embryo development (11). Inducing ER stress with tunicamycin reduced oocyte maturation and inhibited the rate of blastocyst formation by inducing apoptosis (12, 13). The ER stress marker protein, GRP78, was increased in postovulatory aged oocytes, and inhibition of ER stress with the drug, salubrina, can promote embryo development in aged mouse oocytes (14). The Golgi complex has a central roles in trafficking, processing and sorting of membranes and proteins. The Golgi complex undergoes reorganization and redistribution, which is the key feature of cytoplasm maturation during *in vitro* oocyte maturation (15, 16). Lysosomes are dynamic organelles, which are responsible for macromolecule degradation and recycling (17). Inhibition of lysosome function allows damaged DNA to persist, leading to apoptosis, reduced oocyte maturation and embryo development (17, 18). The number and size of lysosomes changed during mouse postovulatory oocyte aging (19). In postovulatory aged porcine oocytes, the potential antioxidant astaxanthin restored signal intensity of organelles including mitochondria, the ER, Golgi apparatus, and lysosomes (20). Previous research indicated that organelle function may be disturbed in aged oocytes, and antioxidants can protect functional organelles during postovulatory oocyte aging. However, the pathways involved in the mechanism of antioxidant-mediated preservation of the optimal distribution and function of organelles during postovulatory oocyte aging remains unknown.

SIRT1, a member of sirtuin family of NAD+− dependent deacetylase, is involved in lifespan extension and anti-aging. SIRT1 participates in multiple biological processes including metabolism, the cell cycle, DNA repair, apoptosis, mitochondrial homeostasis, reducing oxidative stress, and blocking senescence (21). SIRT1 also plays a crucial role in many human conditions such as cardiovascular disease and tumorigenesis (21). The reason for SIRT1’s pleiotropic effects is its large repertoire of deacetylation targets, which not only covers the lysine acetylation of histone proteins H1, H3 and H4, but also includes non-histone targets such as the tumor suppressor p53, forkhead box class O (FoxOs), PPARγ coactivator-1α (PGC1-α), and nuclear factor κB (NF-κB) (22). SIRT1 affects mitochondrial biogenesis by regulating PGC1-α deacetylation (23) and prevents ER stress-induced apoptosis through regulation of eIF2α activities (24). Though SIRT1 has many deacetylation targets and affects the function of mitochondria and ER stress (22, 24, 25), the effect of SIRT1 on the function of ER, Golgi and lysosomes during postovulatory oocyte aging has not been systematically studied.

Luteolin, a natural flavonoid, is contained in a wide range of vegetables and fruits such as celery, parsley, broccoli, scallions, carrots, peppers, cabbages, and apples (26). Luteolin has antioxidant properties, anticancer and anti-inflammatory activities (26, 27), and induces SIRT1 to prevent apoptosis and oxidative stress (28). Also, luteolin is quite heat stable and shows relatively little loss during cooking (29). Luteolin is safe for human use and has been linked to many health benefits (30), but little research has been done on its effects on oocytes and embryo development. Here, we investigated luteolin as an activator of SIRT1, and explored the relationship between luteolin and organelle functions in oocytes during postovulatory aging.

## Materials and methods

Unless otherwise stated, all chemicals were purchased from Sigma-Aldrich (St. Louis, MO, United States).

### Ethics statement

The ICR strain of mice was obtained from the Guangdong Medical Laboratory Animal Center [Cert no. SCXK (Yue) 2022-0002]. The animal procedures used in this study were performed according to the Guide for the Care and Use of Laboratory Animals of the National Research Council. The proper handling of ICR mice during the experimental procedures was approved by the Animal Care and Use Committee of the South China Agricultural University (Approval no: 2022f116).

### Oocyte collection and drug treatment

Sexually mature female ICR mice, in good health, were housed in a ventilated chamber at a controlled temperature (23–25°C) with a 12 h light/dark cycle. Pregnant mare serum gonadotropin (PMSG) and human chorionic gonadotropin (hCG) were injected to induce superovulation. Briefly, 7.5 IU PMSG was intraperitoneally injected into each mouse, and after 48 h 7.5 IU hCG. The cumulus-oocyte complexes (COCs) were collected from the oviductal ampullae 14 to 16 h after hCG injection. For *in vitro* aging, the COCs were cultured in M16 medium (Sigma) with or without luteolin (MCE, HY-N0162) for 12 h in a humidified air. Luteolin (10 mg) was dissolved in 349.4 μL DMSO to give a 100 mM stock that was stored at −80°C. The optimal dose of luteolin was determined by a dose–response experiment (1, 3, 5, 10 μM). The same volume and concentration of DMSO was added to the control group. For the EX-527 treatment, the optimal dose of EX-527 was also determined by dose–response. To separate cumulus complex from the oocytes, the COCs were incubated with 0.1% hyaluronidase in M2 medium. The oocytes obtained from oviductal ampullae at 14 to 16 h after hCG injection were considered as “fresh,” while the oocytes cultured for 12 h were designed as “aged.”

### In vitro fertilization

The IVF procedure was performed according to methods detailed in a previous study (5). Mouse sperm was harvested from the deferent duct and cauda epididymides in human tubal fluid (HTF). The sperm were incubated for 1 h in HTF medium for capacitation to occur, and added into HTF containing the oocytes. After 5–6 h later, fertilized embryos with two pronuclei were selected, washed three times with KSOM+AA medium (Millipore, Billerica, MA, United States), and incubated in KSOM+AA medium in humidified air (37°C, 5% CO2). The ratio of 2-cell, embryos to blastocysts was measured after incubation for 24 h, 4.5 d and 5.5 d.

### Quantitative real-time PCR

The procedure of Quantitative real-time PCR (qRT-PCR) was applied as previously described (25). Briefly, 30 oocytes were dissolved in Cell-to-SignalTM Lysis Buffer (Ambion) and cDNA was directly synthesized according to the protocol of SuperScript^®^ III CellsDirect cDNA Synthesis kit (Thermo Fisher). qRT-PCR was applied with the kit of TB Green^®^ Premix Ex Taq™ (TaKaRa, Beijing, China) on an Applied Biosystems™ QuantStudio™ 5 (Thermo Fisher Scientific). The method of 2-ΔΔCT was used to determine the RNA level. The sequences of Sirt1 and Gapdh primer are listed in Supplementary Table S1. Each reaction was carried out three times with three independent replicates.

### Immunofluorescence

The procedure of Immunofluorescence was carried out as previously described (5). Briefly, the oocytes, fixed with 4% paraformaldehyde (Byotime), were punched with 0.5% Triton X-100 (Byotime) for 10 min, and then globally blocked in QuickBlock™ Blocking Buffer for Immunol Staining (Byotime) for 1 h. The oocytes were cultivated for 8 h at 4°C in first antibodies diluted in QuickBlock™ Primary Antibody Dilution Buffer (P0262, Byotime). After washed thoroughly in PBS, the samples were stained with the diluted Alexa Fluor 488 or 555 goat anti-rabbit or anti-mouse IgG (H + L) (1:600, Invitrogen, Carlsbad, CA, United States) for 2 h at room temperature, and then stained with DAPI (C1006, Beyotime) for 3 min. The antibodies are listed as follows: rabbit anti-SIRT1 (ab189494, 1:100, Abcam), mouse anti-α-tubulin (ab7291, 1:600, Abcam), rabbit anti-α-tubulin (acetyl K40) (ab179484, 1:500, Abcam), rabbit anti- Anti-p53 (acetyl K382) (ab75754, 1:200, Abcam)rabbit anti-GRP78 (ab108615, 1:100, Abcam), and rabbit anti-GOLPH3 (AP6024, 1:100, Bioworlde), rabbit anti-LAMP1 (ab208943, 1:100, Abcam). Finally, the signals were examined with a Zeiss Axio Observer D1 microscope (Carl Zeiss, Inc., Thornwood, NY). The procedure of staining and settings of microscope were the same for each group.

### ROS measurement and Annexin-V assay

The intercellular ROS was determined by Reactive Oxygen Species Assay Kit (Yeason, ShangHai, China). We used DCFH-DA (1:1000) diluted in M2 medium to incubate the different groups of living oocytes for 30 min. The Annexin V-FITC Apoptosis Detection kit was used to detect apoptosis. The living oocytes washed with PBS were incubated for 30 min in Annexin V-FITC combination buffer (195 μL) added with Annexin V-FITC (5 μL). Finally, the staining of oocytes were captured with a Zeiss Axio Observer D1 microscope (Carl Zeiss, Inc., Thornwood, NY). The procedure of staining and settings of microscope were keeping same for each group.

### Rhodamine phalloidin staining

The procedure for rhodamine phalloidin staining followed the protocol provided by the Rhodamine Phalloidin kit (HY-K0903, MCE, China). Briefly, the oocytes were fixed with 4% paraformaldehyde (Byotime) for 5 min, followed by permeabilization with 0.5% Triton X-100 (Byotime) for 10 min. After three washes with PBS, the samples were incubated in M2 medium containing rhodamine phalloidin (1:1000) for 30 min away from light. Subsequently, the samples were washed three times with PBS and visualized using a Zeiss Axio Observer D1 microscope (Carl Zeiss, Inc., Thornwood, NY).

### Mitochondria and ER detection

In order to detect mitochondrial distribution, the denuded living oocytes were moved into Mito-Tracker green (200 nM, Beyotime, Shanghai, China) diluted in M2 medium for 30 min at 37°C in dark. The mitochondrial membrane potential was measured with Mitochondrial Membrane Potential Assay Kit with TMRE (C2001S, Beyotime); the tetramethylrhodamine, ethyl ester (TRME) can gather in matrix of polar mitochondria with orange fluorescence, and there is no TMRE accumulation in lower membrane potential of mitochondria. The cumulus-denuded oocytes were stained with TMRE (1:1000) for 20 min away from light. For ER staining, it is 30 min for the living oocytes incubating in ER tracker green (C1042S, 1:1000, Beyotime). For staining DNA of living oocytes, the oocytes were cultured with Hoechst 33342 (C1025, Beyotime) for 10 min. Finally, the samples were scanned with a Zeiss Axio Observer D1 microscope (Carl Zeiss, Inc., Thornwood, NY). It was keeping the same for the procedure of staining and settings of microscope in each group.

### Golgi complex detection

The Golgi apparatus detection was applied according to previous studies (31). For detecting the distribution of Golgi apparatus, the zona pellucida was removed by incubating with 1% pronase (Sigma) for 10 min. The living oocytes without zona pellucida were cultured in M2 medium with Golgi-Tracker Red at 4°C for 30 min, and then incubated in fresh M2 medium at 37°C for 30 min. Finally, different groups of oocytes were incubated with Hoechst 33342 for 10 min to stain DNA. Finally, the signals for each group were captured with a Zeiss Axio Observer D1 microscope (Carl Zeiss, Inc., Thornwood, NY). The procedure of staining and settings of microscope were the same for each group.

### Western blotting

Western blotting was used to validate the Immunofluorescence results of SIRT1 and GOLPH3. Briefly, 200 oocytes for each group were separately dissolved with the buffer of RIPA (Beyotime, P0013B). The samples were loaded on 12% acrylamide gels, and then transferred to PVDF membranes (Millipore,Bedford, MA, United States) with semi-dry transfer. After blocked with QuickBlock™ Blocking Buffer for Western Blot (P0252, Beyotime) for 1 h, the PVDF membranes with proteins were cultured with primary antibodies such as rabbit anti-SIRT1 (ab189494, Abcam), GAPDH Mouse mono antibody (AC002, Abclonal), rabbit anti-GOLPH3 (AP6024, Bioworld). After washed thoroughly, the membranes were stirred in horse radish peroxidase conjugated Goat Anti-Rabbit or Mouse IgG (H + L) (Abclonal) for 1 h at room temperature. The bands of SIRT1 and GOLPH3 were analyzed and normalized to GAPDH with ImageJ software.

### Statistical analysis

Each experiment was repeated three times. *n* is as the number of samples examined. Tow comparisons were analyzed using independent *t* tests with the GraphPad Prism 5 software (GraphPad, San Diego, CA). The data are shown as mean ± SEM. the signals was analyzed with ImageJ and the histogram was made with GraphPad Prism 5 software. The statistically significant was accepted with *p* < 0.05.

## Results

### Luteolin promotes the development of aged oocytes after fertilization

**Figure 1:**
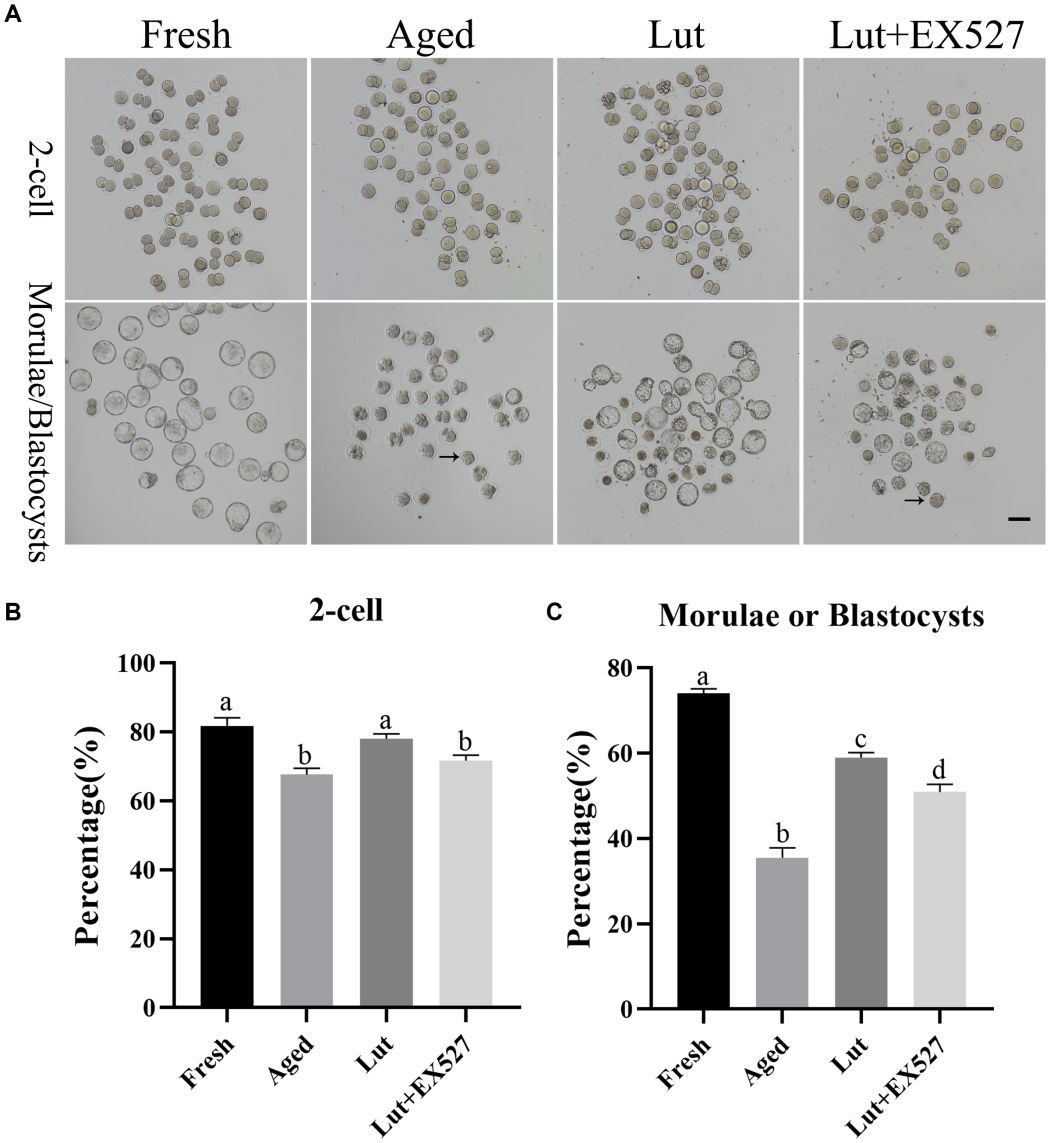
Luteolin improving and EX527 inhibiting the development of embryos derived from aged oocytes after fertilization. **(A)** The typical figures of embryos at 2-cell and blastocyst stages from fresh, aged, luteolin (lut, 5 μM), and Lut (5 μM) + EX527 (0.5 μM) groups (*n* = 120 for each group). Arrow, morula; Scale bar, 100 μm. **(B,C)** The rates of 2-cell and blastocyst (day 4.5) calculated from different groups, respectively, (*n* = 120 for each group). Different superscripts of a, b, c, d mean differences at *p* < 0.05.

To determine the optimal dose of luteolin for delaying postovulatory oocyte aging, the oocytes were incubated for 12 h with luteolin at a range of concentrations: 1, 3, 5, and 10 μM. The percent fertilization was markedly reduced in the aged groups compared with the fresh groups (64.33 ± 3.40% vs. 81.80 ± 3.25%, *p* < 0.05; Figures 1A,B; Supplementary Table S2), but luteolin treatment significantly increased the rate of fertilization during postovulatory aging (76.12 ± 3.13% vs. 64.33 ± 3.40%, *p* < 0.05; Figures 1A,B; Supplementary Table S2). The fertilized embryos were cultured in KSOM medium for 4.5 days, and the rate of blastocysts formation was calculated. The percent of blastocysts was significantly low-er in the aged groups compared with fresh ones (35.50 ± 3.26 vs. 74.05 ± 1.46%, *p* < 0.05; Figures 1A,C; Supplementary Table S2). About 70% embryos (morula/(morula + blastocyst)) are still at morula stage at 4.5 days after fertilization in aged groups, while in the fresh and luteolin-treated groups most were at the blastocyst stage (Figure 1A; Supplementary Table S2). Incubation with 5 μM luteolin gave the highest percentage of blastocysts in the treated groups, and the difference with the aged groups was significantly (61.06 ± 3.62% vs. 35.50 ± 3.26%, *p* < 0.05; Figures 1A,C; Supplementary Table S2); so 5 μM luteolin was used in the subsequent research.

### SIRT1 controls the development potential of embryos

**Figure 2:**
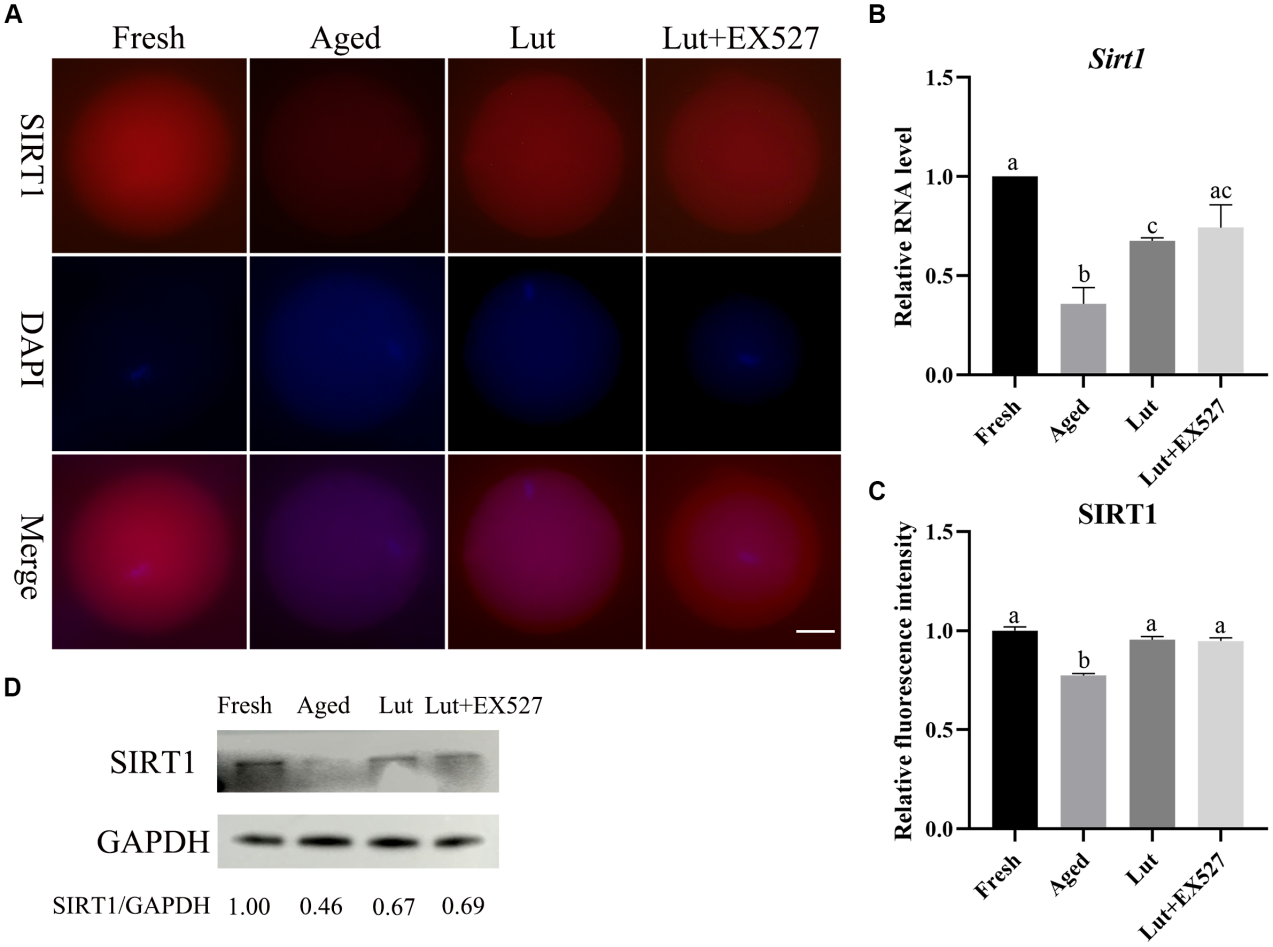
The expression of SIRT1 in oocytes from fresh, aged, luteolin, and Lut + EX527 groups. **(A)** The representative figures of SIRT1 in oocytes from fresh, aged, luteolin (Lut, 5 μM), and Lut + EX527 (0.5 μM) groups. Scale bar, 30 μm. **(B)** The qPCR results of *Sirt1* from four different groups. **(C)** The relative intensity of SIRT1 signals analyzed in oocytes from four different groups (*n* = 20 for each group). **(D)** The result of western blot and analysis result of SIRT1 from four different groups (*n* = 200 for each group). Difference of a, b, c indicates *p* < 0.05.

SIRT1 has anticancer and can enhance longevity, and it is effective in preventing postovulatory oocyte aging (3, 25). Luteolin is contained in many fruits and vegetables, where it acts to prevent oxidative stress and it appears to be an activator of SIRT1 (32). To determine whether luteolin’ effect in delaying oocyte aging was mediated through the regulation of SIRT1 activity, the expression levels of SIRT1 were measured under various conditions. The mRNA and protein levels of SIRT1 were significantly lower in aged groups (*p* < 0.05, Figures 2A–C), which is in line with previous studies (3); but, after luteolin treatment, the mRNA and protein levels of SIRT1 significantly increased compared with non-treated aged groups (*p* < 0.05, Figures 2A–C). This indicated that luteolin prevented the decrease in SIRT1 during postovulatory oocyte aging.

EX-527, an inhibitor of SIRT1, was tested in combination with luteolin to investigate the function of SIRT1 in embryo development. First, the optimal dose of EX-527 for inhibiting SIRT1 activity was determined by dose–response experiment (0, 0.1, 0.2, 0.5, and 1 μM EX-527). When treated exclusively with EX527, the formation rate of blastocysts was observed to decrease in the groups exposed to 0.2 μM, 0.5 μM, and 1 μM concentrations of EX527. Among these groups, the lowest rate of blastocyst formation was recorded in the 0.5 μM EX527 group. However, this rate was not found to be significantly different when compared to the aged groups (*p* < 0.05; Supplementary Figure S3). When co-treated with Lut and EX527, the rate of blastocyst formation significantly reduced in the 5 μM Lut + 0.5 μM EX527 groups compared with the Lut-only group (50.41 ± 3.12% vs. 63.64 ± 1.73%, *p* < 0.05; Figure 1C; Supplementary Figure S2; and Supplementary Table S4). In addition, at 4.5 days after fertilization, about 60% of embryos (morula/ (morula + blastocyst)) stayed at morulae stage in the 5 μM Lut + 0.5 μM EX527 groups (Supplementary Figure S2A; Supplementary Table S4), and they will develop to blastocyst at 5.5 days (Supplementary Figure S2B; Supplementary Table S4). Most aged embryos were at the morula stage at 4.5 days, whereas they should be at blastocyst stage, and inhibition of SIRT1 delayed the embryo development after fertilization (Figure 1A;Supplementary Figure S2). Clearly, SIRT1 affects the speed of embryo development after fertilization.

There is no significant difference in the mRNA and protein level of SIRT1 in the Lut + EX527 groups compared with the Lut-only group (Figures 2A–C). However, the rate of blastocyst formation was significantly lower in the Lut + EX527 group compared with the Lut-only group (*p* < 0.05, Figures 1A,C; Supplementary Table S4), supporting a role for EX527 in embryogenesis. The western blotting results showed that the level of SIRT1 decreased in aged groups and increased in Lut and Lut + EX527 groups (Figure 2D), in agreement with the results of SIRT1 staining. We conclude that inhibition of SIRT1 significantly prevented the development of competence of aged oocytes promoted by luteolin.

### Luteolin affects ROS accumulation, apoptosis, and F-actin distribution

**Figure 3:**
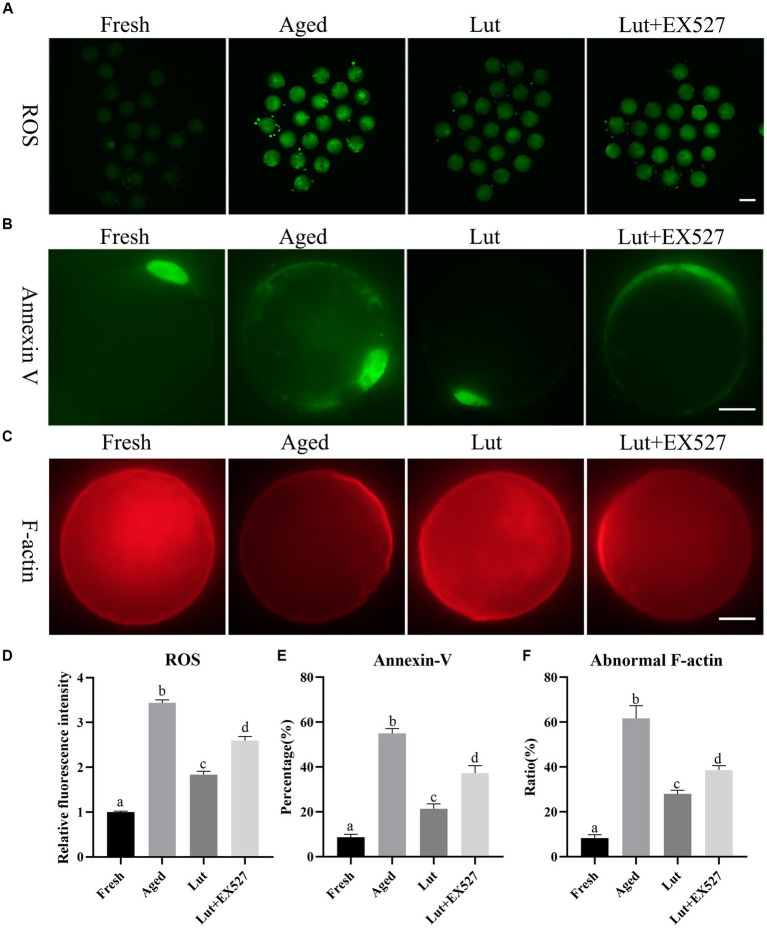
Effects of luteolin on the ROS accumulation, apoptosis and F-actin distribution. **(A)** The typical images of ROS in oocytes from fresh, aged, lut, and Lut + EX527 groups. Scale bar, 100 μm. **(B)** Analysis relative intensity of signals of ROS in oocytes from different groups (*n* = 20 for each group). **(C)** The images of annexin-V presented from different groups. Scale bar, 30 μm. **(D)** Analysis and calculation of ratio of annexin-V signals in different groups (*n* = 80 for each group). **(E)** The representative figures of rhodamine phalloidin staining in fresh, aged, lut, and Lut + EX527 oocytes. Scale bar, 30 μm. **(F)** Analysis the proportion of abnormal F-actin distribution from different groups (*n* = 60 for each group). Different superscripts of a, b, c, d mean differences at *p* < 0.05.

High ROS levels can damage mitochondria and induce apoptosis in cells, and they are regarded as the main cause of oocyte aging (33). In line with previous results, the ROS level significantly increased in aged group relative to the fresh group (*p* < 0.05; Figures 3A,D) (10). Incubation with Luteolin significantly reduced the ROS level compared with aged groups (*p* < 0.05; Figures 3A,D). When oocytes were co-treated with Lut + EX527, the level of ROS increased again compared with the Lut-only group (*p* < 0.05; Figures 3A,D).

The apoptosis reagent, annexin V-FITC binds to phosphatidylserine, which is located at the outer membrane surface of cells at the early stages of apoptosis. There were no annexin V signals on fresh oocytes, while intense annexin V fluorescence was seen surrounding aged oocytes (Figure 3B). The percentage of annexin V-positive oocytes was significantly higher in the aged group relative to fresh oocytes (54.99 ± 3.13% vs. 8.78 ± 1.75%, *p* < 0.05; Figure 3E). Moreover, Luteolin significantly reduced the rate of apoptosis relative to that in aged oocytes (21.44 ± 2.99% vs. 54.99 ± 3.13%, *p* < 0.05; Figure 3E). The apoptosis rate was 37.3% in the Lut + EX-527 group, which was significantly higher than the Lut-only group (*p* < 0.05, Figure 3E).

Rhodamine phalloidin was used to visualize the distribution of microfilament cytoskeleton in cells by binding to F-actin. The staining results with rhodamine phalloidin revealed that F-actin exhibited an even distribution at the edge of oocytes, but its distribution was partially disrupted in aged oocytes (Figure 3C). The incidence of abnormal F-actin distribution was significantly higher in aged oocytes compared to fresh ones (61.63 ± 8.10% vs. 8.33 ± 2.15%, *p* < 0.05; Figure 3F). Treatment with luteolin significantly reduced the percentage of abnormal F-actin distribution in comparison to the aged group (28.06 ± 2.19% vs. 61.63 ± 8.10%, *p* < 0.05; Figure 3F). However, when co-treated with EX527 and luteolin, the percentage of abnormal F-actin distribution was significantly higher compared to the Lut group (38.65 ± 2.78% vs. 28.06 ± 2.19%, *p* < 0.05; Figure 3F). Based on these findings, we conclude that luteolin can mitigate the detrimental effects of aging, such as ROS accumulation, apoptosis, and altered F-actin distribution, by regulating the activity of SIRT1.

### Luteolin affects spindle morphology as well as the acetylation of α-tubulin and p53

**Figure 4:**
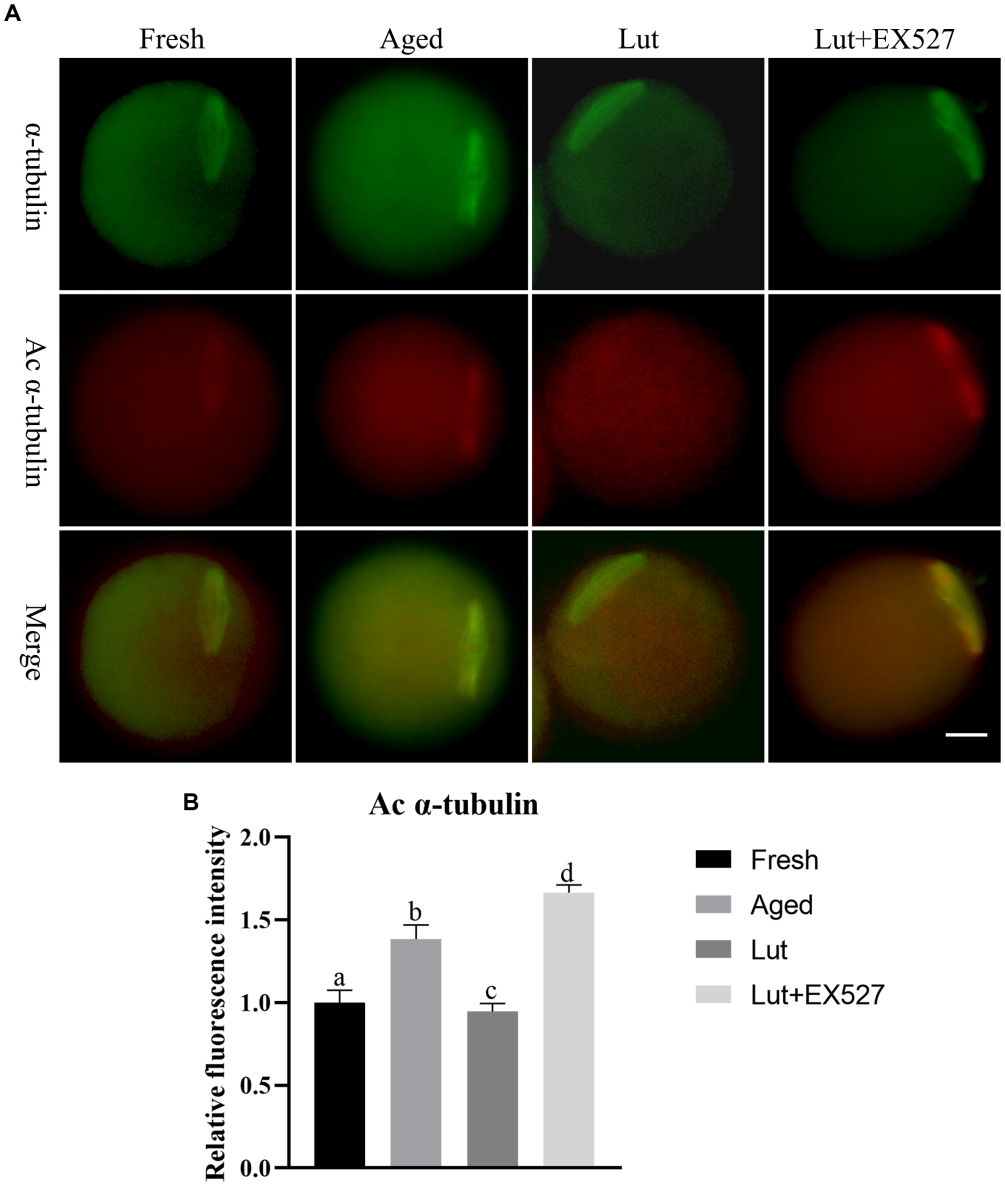
Effects of luteolin on the level of a, c α-tubulin. **(A)** The representative images of α-tubulin and a, c α-tubulin in fresh, aged, lut, and Lut + EX527 oocytes. **(B)** Analysis the intensity of a, c α-tubulin from four different groups (*n* = 20 for each group).

An intact spindle is critical for when the chromosomes enter the blastomere stage, to avoid aneuploidy during cell division. However, the typical barrel-shaped spindles with chromosomes in fresh oocytes are elongated or disturbed in the aged group (Figure 4A; Supplementary Figure S1A). Lut incubation can maintain the normal barrel-shaped spindle and avoid the aging effect (Figure 4A; Supplementary Figures S1A,B). The participation of SIRT1 in this process is supported by the observation that the normal spindle structure is disrupted by EX-527, and the percentage of abnormal spindles increased significantly in the Lut + EX-527 group compared with the Lut-only group (54.55 ± 3.71% vs. 28.20 ± 2.80%, *p* < 0.05; Supplementary Figure S1B).

The previous study indicated that the acetylation of α-tubulin (a, c α-tubulin) increases during postovulatory oocyte aging (34). As SIRT1 is involved in the de-acetylation of α-tubulin (35), we investigated the intensity of a, c α-tubulin signals in fresh and aged oocytes. The staining results showed a significant increase in the intensity of a, c α-tubulin signals in aged oocytes compared to fresh oocytes (*p* < 0.05; Figures 4A,B). To further explore the potential role of SIRT1 in regulating a, c α-tubulin levels, we treated oocytes with luteolin. Luteolin treatment significantly decreased the intensity of a, c α-tubulin signals, suggesting its ability to counteract the aging-induced spindle abnormalities (*p* < 0.05; Figures 4A,B). Notably, the effect of luteolin on a, c α-tubulin was reversed when combined with EX-527, an inhibitor of SIRT1 (*p* < 0.05; Figures 4A,B).

The present study also examined p53 lysine-382, another target of SIRT1 deacetylation. The intensity signals of p53 acetylation (a, c p53) were found to be significantly higher in aged groups compared to fresh ones (*p* < 0.05; Supplementary Figure S3). Luteolin led to a significant decrease in a, c p53 intensity when compared to the aged groups. However, this effect of luteolin on a, c p53 was reversed when SIRT1 was inhibited with EX527 (*p* < 0.05; Supplementary Figure S3). These results demonstrated that luteolin mitigates spindle abnormalities and restores the abnormal levels of a, c α-tubulin and p53 by modulating SIRT1 activity.

### Luteolin affects the distribution and function of mitochondria

**Figure 5:**
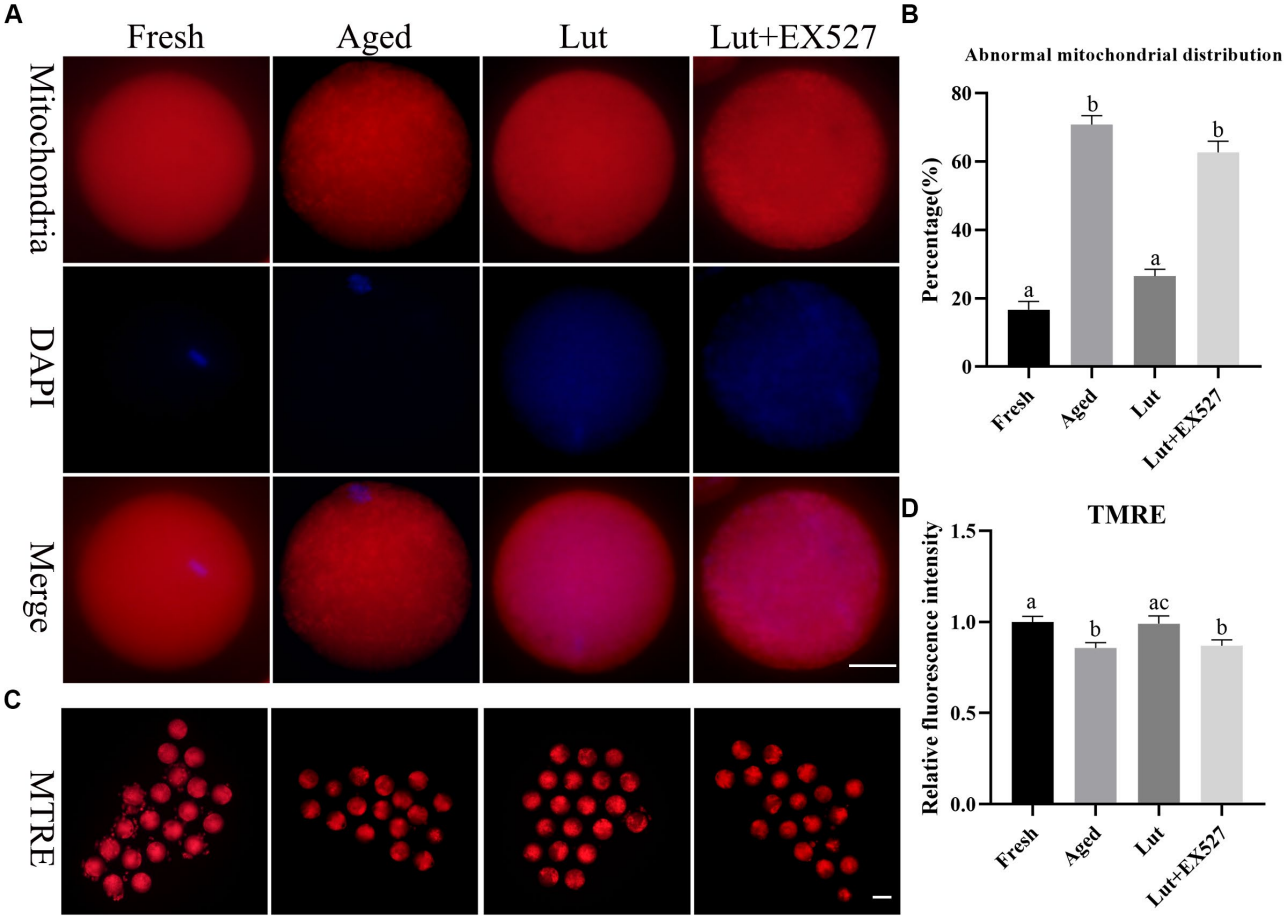
Effects of luteolin on mitochondrial distribution and function. **(A)** The typical figures of mitochondria in oocytes from fresh, aged, lut, and Lut + EX527 groups. Scale bar, 30 μm. **(B)** Histogram showing the proportion of abnormal distribution of mitochondria from different groups (*n* = 80 for each group). **(C)** The representative figures of TMRE in oocytes separately from different groups. Scale bar, 100 μm **(D)** Quantification analysis of the signals of TMRE in each oocyte from different groups (*n* = 20 for each group). Difference of a, b indicates *p* < 0.05.

Mitochondrial homeostasis is critical for preserving oocyte quality and the developmental potential of embryos (36). The mitochondria, stained with Mito-Tracker green, were evenly distributed in the cytoplasm of fresh oocytes, while they were gathered into clumps in aged oocytes (Figure 5A). The abnormal distribution of mitochondria significantly increased in aged oocytes compared with fresh ones (70.81 ± 3.76% vs. 16.67 ± 3.40%, *p* < 0.05; Figure 5B), but was significantly reduced by treatment with Lut (26.49 ± 2.83% vs. 70.81 ± 3.76%, *p* < 0.05; Figure 5B). However, the percentage of abnormal mitochondria was significantly increased again after inhibiting SIRT1 with EX527 (62.69 ± 4.61% vs. 26.49 ± 2.83%, *p* < 0.05; Figure 5B). Furthermore, the mitochondrial membrane potential, which indicates normal mitochondrial function, was reduced during postovulatory oocyte aging (10). In the present study, the membrane potential of mitochondria was significantly lower in aged groups compared with counterparts (*p* < 0.05; Figures 5C,D). When SIRT1 was activated by Lut, however, the low membrane potential was returned to normal, and this effect was reversed by inclusion of the SIRT1 inhibitor (*p* < 0.05; Figures 5C,D). These results indicated that SIRT1 regulated the distribution and function of mitochondria in oocytes during postovulatory oocyte aging.

### Luteolin affects the distribution and function of endoplasmic reticulum

**Figure 6:**
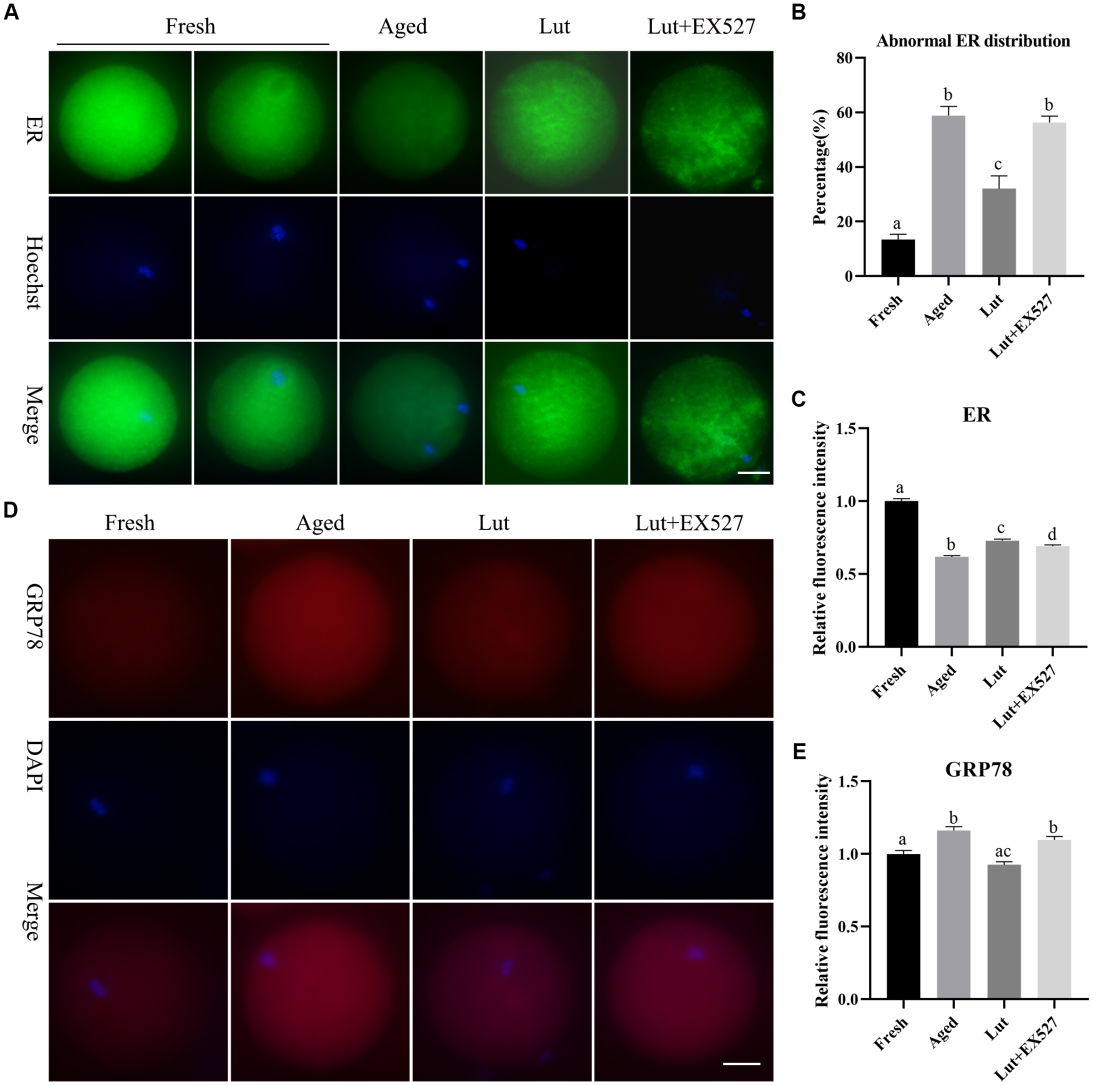
Effects of luteolin on the distribution and function of ER during postovulatory oocyte aging. **(A)** The typical images showing the staining results of ER in oocytes from fresh, aged, lut, and Lut + EX527 groups. Scale bar, 30 μm. **(B)** The percentage of abnormal ER distribution in different groups were analyzed (*n* = 60 for each group). **(C)** Quantification analysis of the intensity of ER staining in each oocyte from different groups (*n* = 20 for each group). **(D)** Histogram showing the representative figures of GRP78 in oocytes derived from different groups. Scale bar, 30 μm. **(E)** Analysis of relative intensity of GRP78 in oocytes from different groups (*n* = 15 for each group). Difference of superscripts indicates *p* < 0.05.

The endoplasmic reticulum is responsible for correct protein folding and translocation. In order to measure the effect of Lut/SIRT1 on the distribution of ER, the fluorescent marker, the ER-Tracker Green was used to stain the oocytes. The ER was evenly distributed in the cytoplasm with surrounding DNA in fresh oocytes, however, in the aged group, the ER was gathered around the edges of the cytoplasm and showed lower staining intensity (Figures 6A,B). The abnormal ER distribution in the aged group was significantly less (32.04 ± 6.68% vs. 58.81 ± 4.77%, *p* < 0.05; Figure 6B) and the intensity of ER signals significantly increased in the oocytes incubated with Lut (*p* < 0.05; Figure 6C). Inhibition of SIRT1 with EX527, resulted in a significant increase in abnormal ER and the fluorescent signals was lower compared with the Lut group (32.04 ± 6.68% vs. 56.27 ± 3.36%, *p* < 0.05; Figures 6B,C).

If the ER environment is disturbed by oxidative stress, DNA damage and calcium depletion, the ER stress could result in accumulation of unfolded or misfolded proteins in the ER (37). The ER stress protein GRP78 was stained to detect the ER stress in oocytes. The immunofluorescence results showed that GRP78 was significantly increased in aged oocytes compared with fresh ones (*p* < 0.05; Figures 6D,E), similar to the results of previous studies (14). The intensity of GRP78 was significantly diminished in the Lut group compared with the aged group (*p* < 0.05; Figure 6E). When SIRT1 was inhibited with EX527, the GRP78 stress protein level increased again compared with the Lut group (*p* < 0.05; Figure 6E). Thus, SIRT1 manages the distribution and function of the ER during postovulatory oocyte aging.

### Luteolin modulates the distribution and function of Golgi complex

**Figure 7:**
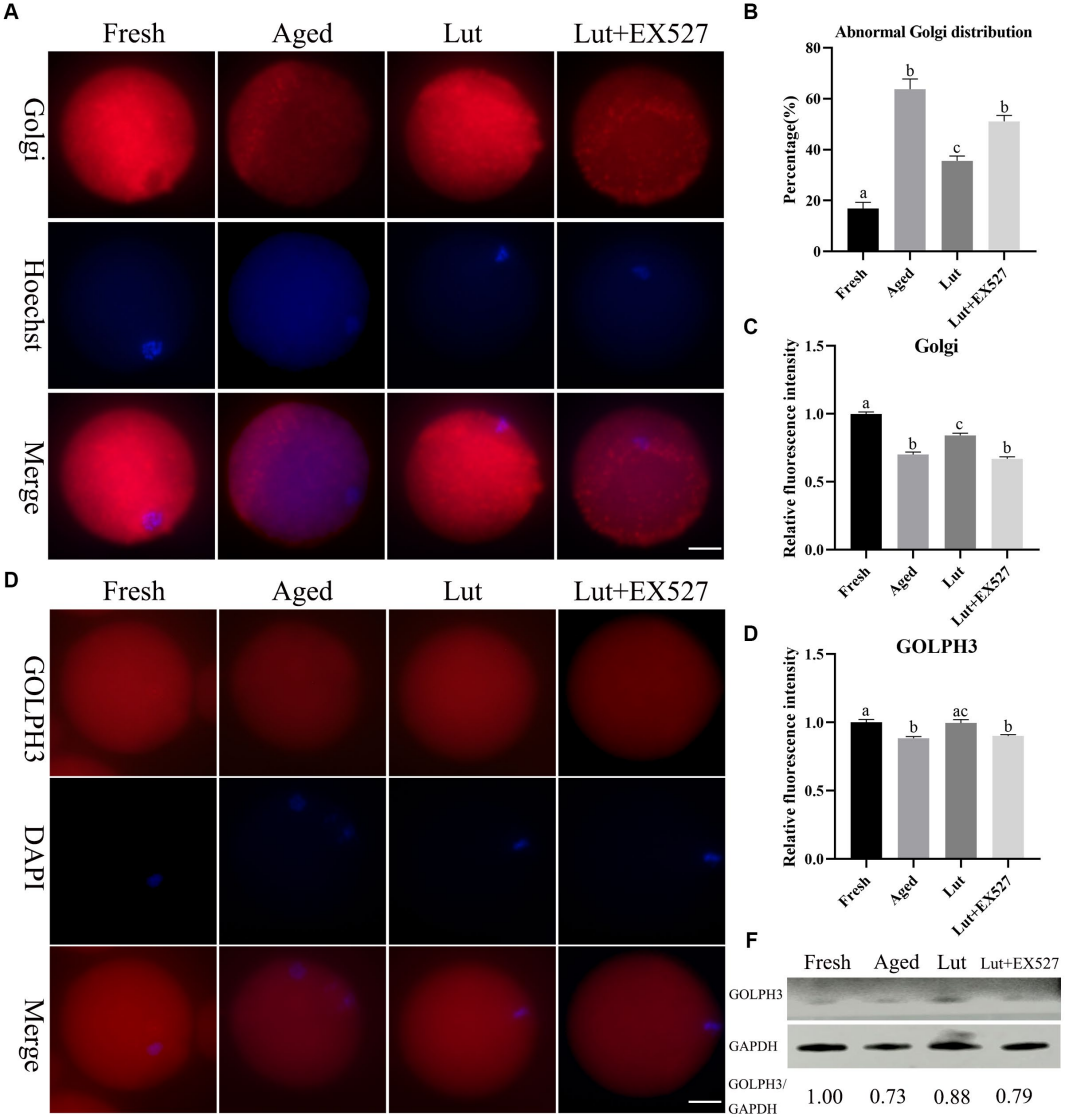
The impact of luteolin on Golgi apparatus. **(A)** The representative pictures showing the results of staining Golgi apparatus in oocytes from fresh, aged, lut, and Lut + EX527 groups. Scale bar, 30 μm. **(B)** Calculation of the ratio of abnormal Golgi apparatus distribution in oocytes from different groups (*n* = 90 for each group). **(C)** Histogram showing the results of relative staining intensity of Golgi apparatus in each oocyte from different groups (*n* = 20 for each group). **(D)** The typical figures of GOLPH3 in oocytes derived from different groups. Scale bar, 30 μm. **(E)** Quantification calculation of the intensity of GOLPH3 in oocytes from different groups (*n* = 20 for each group). **(F)** The results of western blot determining the protein level of GOLPH3 from different groups (*n* = 200 for each group). Difference of superscripts indicates *p* < 0.05.

The Golgi apparatus is responsible for modifications such as glycosylation and sulfation of newly synthesized proteins transported from the ER (38). The Golgi was found to be associated with spindle assembly and division during *in vitro* maturation of oocytes (39). The Golgi-Tracker Red kit was used to determine the distribution of Golgi. The Golgi apparatus was punctate and concentrated in the cytoplasm of fresh oocytes, while it seemed to disappear in aged oocytes (Figure 7A). When Lut was included in the incubation medium during postovulatory oocyte aging, the abnormal Golgi distribution was significantly reversed (35.66 ± 2.60% vs. 63.69 ± 5.77%, *p* < 0.05; Figure 7B) and the staining intensity was much greater compared with aged groups in the absence of Lut (*p* < 0.05; Figure 7C). After treatment with EX527, the rate of abnormal distribution of punctate Golgi was significantly higher (51.08 ± 3.37% vs. 35.66 ± 2.60%, *p* < 0.05; Figure 7B) and the intensity of Golgi signals significantly decreased compared with the Lut groups (*p* < 0.05; Figure 7C).

Golgi phosphoprotein 3 (Golph3) is responsible for Golgi organization and protein transportation from Golgi to the plasma membrane (40). GOLPH3 was evenly distributed in the cytoplasm of fresh oocytes, but the intensity of GOLPH3 was significantly lower in the aged oocytes compared with fresh ones (*p* < 0.05; Figures 7D,E). Luteolin up-regulated GOLPH3 but this significantly reversed by EX-527 treatment (*p* < 0.05; Figures 7D,E). The western blot results also indicated that GOLPH3 decreased in aged oocytes, increased with Lut treatment and then decreased when EX527 was included (Figure 7F). Thus, based on these results, we conclude that luteolin directs the distribution of the Golgi and increases the expression of GOLPH3 by regulating SIRT1 to regulate the function of the Golgi apparatus.

### Luteolin directs the function of lysosomes

**Figure 8:**
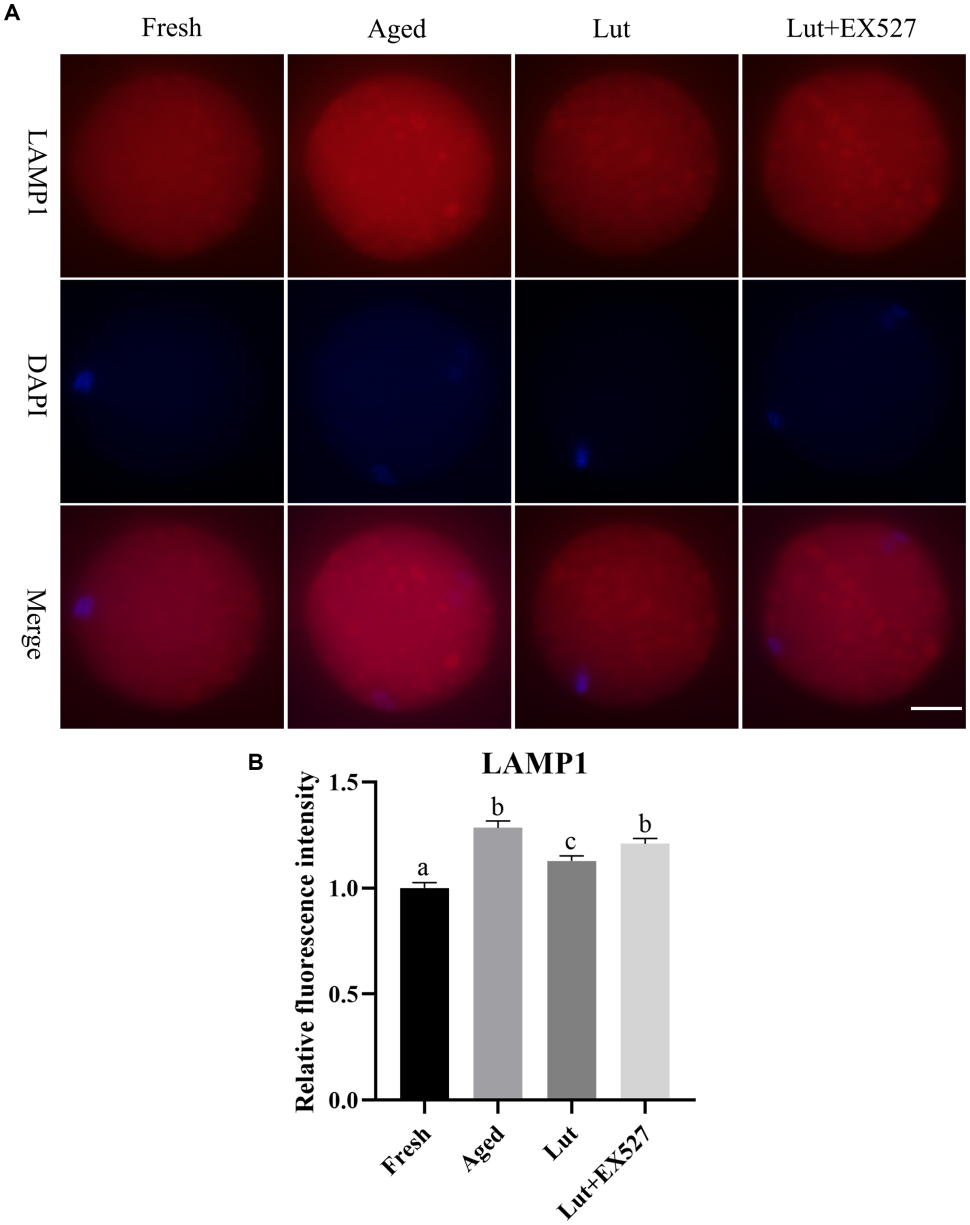
The impact of luteolin on lysosomes protein LAMP1. **(A)** The representative pictures of LAMP1 in oocytes derived from fresh, aged, lut, and Lut + EX527 groups. Scale bar, 30 μm. **(B)** Analysis of relative intensity of LAMP1 signals in oocytes from different groups (*n* = 20 for each group). Difference of superscripts indicates *p* < 0.05.

Lysosomes are involved in the process of degrading and recycling cellular waste (41). LAMP1, which is a key component of lysosomes, was used as a marker to identify lysosomes (19). The staining of LAMP1 showed that lysosomes were larger in aged oocytes than in fresh ones, and the intensity of LAMP1 was significantly increased in the aged group (Figures 8A,B); in agreement with previous study (19). However, activating SIRT1 with Lut during postovulatory aging significantly decreased the intensity of LAMP1 compared with the aged group (Figures 8A,B). Inhibiting SIRT1 activity with EX-527 significantly reduced the intensity of lysosome staining for LAMP1 with the Lut group (Figures 8A,B). The results confirmed that luteolin could regulate the function of lysosomes.

## Discussion

Postovulatory oocyte aging is a potentially damaging factor in human assisted reproduction. SIRT1, as the longevity gene, has been proved to prevent postovulatory oocyte aging (3). In the present study, mouse oocytes were used as the model to investigate the effect of luteolin on the distribution and function of organelles. Luteolin, the activator of SIRT1, significantly prevented postovulatory oocyte aging and increased the blastocyst formation rate after *in vitro* fertilization. Inhibiting SIRT1 activity not only lowered the rate of blastocyst formation, but also unexpectedly delayed the time of embryo development to blastocyst stages. The aging-induced defects including abnormal spindle morphology, ROS accumulation and apoptosis were corrected by luteolin. The distribution and function of organelles such as mitochondria, ER, Golgi and lysosomes were disrupted in aged oocytes, but luteolin was able to restore the normal distribution and function of organelles during postovulatory oocyte aging. EX-527, an inhibitor of SIRT1, reversed the effect of luteolin on attenuating aging-induced abnormalities including spindles, oxidative stress, apoptosis, and organelle function.

It is well known that postovulatory aged oocytes lose development competence and that antioxidants such as melatonin, resveratrol, and quercetin prevent oocyte aging by regulating SIRT1 activities (10, 42, 43). The present research also proved that luteolin could protect oocytes from postovulatory aging. Luteolin prevented aging-induced abnormalities including ROS accumulation, apoptosis, abnormal spindles, and organelle dysfunction and delayed oocyte aging. Luteolin is a flavonoid found in vegetables. It has contrary function as an antioxidant depending on concentrations. When the Lut concentration was less than 10 μM, it inhibited ROS production and prevented chemical toxicity by activating SIRT1 (28, 44). However, at a concerntration of 40 or 50 μM, Lut would induced ROS production and DNA damage with mitochondrial dysfunction, and this led to the death of cancer cells (45, 46). Thus, the proper dose of luteolin is crucial for determining a particular cell fate. The 5 μM Lut used here efficiently delayed postovulatory oocyte aging, and there were no negative effects as found in the present study.

SIRT1, as a member of the NAD+ protein family, is involved in diverse biological events, including life-span extension, cellular senescence, age-related disorders, obesity-associated disease, heart disease, inflammation, and cancer (47, 48). SIRT1 can remove acetyl groups from variety targets. SIRT1 deacetylates the acetyl of H3K4, H3K9 and H4K16 to regulate the transcription of tumor-related genes (49–51). SIRT1 can prevent diseases by directly deacetylating p53, SMAD3, and β-catenin (47, 48). SIRT1 regulates the forkhead O family (FOXO) of transcription factors that control oxidative stress, and this is one mechanism of how SIRT1 delays aging and extends lifespan (52, 53). SIRT1 directly deacetylates histone targets to control transcription and deacetylates non-histone targets to regulate protein activities and signal pathways.

SIRT1 has been proved to regulate mitochondrial function, ER stress and lysosomal function. SIRT1 is implicated in metabolism and mitochondrial biogenesis by deacetylating PGC-1α (22), also participates in turnover of damaged mitochondria by mitophagy (22). SIRT1 protects cells and organs from ER stress damage through deacetylation of transcription factors (24, 54). SIRT1 controls lysosomal acidification to regulate breast cancer invasion (55). The present study not only showed that the function of mitochondria, ER and lysosome was controlled by SIRT1, but that the Golgi apparatus function was also directed by SIRT1 in oocytes. Given that the organelles such as mitochondria, ER, Golgi, and lysosomes are the functional places of metabolism, protein synthesis, post-transcriptional modification and turnover of cellular waste (22, 55, 56), luteolin may indirectly affect protein synthesis, modification and turnover by controlling organelle function. This work extends the knowledge about luteolin and SIRT1 function.

Both the function and distribution of organelles was controlled by SIRT1 in oocytes. Autophagy and ER stress are the focus of many studies (22, 56), and the distribution of organelles has been largely ignored. The orderly distribution of organelles in oocytes, as the largest cells in mammals, is important for the mitochondria to be evenly dispersed in the cytoplasm and the ER to surround the chromosomes in MII oocytes (31). When the oocytes were damaged by chemicals or aging, the normal distribution of organelles, especially mitochondria, was disrupted (5, 31). The abnormal distribution of organelles is usually associated with dysfunction, and the distribution of organelles would be an important marker of oocyte quality. In previous studies, only the distribution of mitochondria was found to be altered by SIRT1 (3, 25), so it is important to show that SIRT1 affects the distribution of organelles including the ER, the Golgi complex, and lysosomes. Many proteins affect organelle distribution. RAB8A GTPase and Ral GTPase alter the distribution of the Golgi apparatus in oocytes (57, 58). Rab6a is involved in the organization of the ER, but not the Golgi during mouse oocyte maturation (59). The SCMC (subcortical maternal complex), comprises multiple proteins, and is required for the function and distribution of mitochondria and ER in oocytes (60, 61). Therefore, SIRT1 may target these proteins to control the distribution of organelles in oocytes.

It is well known that defects in organelles can be induced by environmental endocrine disruptors or mycotoxins during oocyte maturation (1, 62, 63). Nonylphenol and nivalenol impair oocyte quality by disrupting spindle organization and organelle function during oocyte aging (1, 63). Fumonisin B1, bisphenol A and aflatoxin B1, specific toxic contained in the environment and in food, disturb the function and distribution of organelles such as mitochondria, ER complex, Golgi apparatus and lysosomes in oocytes (62, 64, 65). Infertility and sterility may be caused by a toxic environment that reduces the quality of oocytes. Here, we also noted defects in organelles including mitochondria, ER, Golgi apparatus, and lysosomes during oocyte aging. These results indicate that the organization of organelles can easily to be damaged by adverse effects, and fortunately this can be efficiently reversed by luteolin treatment.

Here for the first time, we showed that SIRT1 could regulate the speed of embryo development to the blastocyst stage after fertilization. During the morula to blastocyst transformation, the Hippo pathways and polarity proteins such as Par3 and Par6 are involved in the process (66). However, there is no evidence that SIRT1 is involved in the process of morulae formation and blastocyst transformation, and the mitochondria, ER, Golgi apparatus, and lysosomes have not been reported to participate in the morula to blastocyst transformation. Moreover, it is should be noted that inhibiting SIRT1 in oocytes can affect the morula to blastocyst transformation about 3–5 days later. More studies need to done to identify the unknown deacetylation targets of SIRT1 affecting the development speed of embryos.

The present study showed that luteolin, which is contained in fruits and vegetables, delayed postovulatory oocyte aging, and controlled the distribution and function of organelles by increasing SIRT1 activities during postovulatory oocyte aging. The present results extend our knowledge of luteolin and SIRT1 function, and this is beneficial for promoting the success of artificial assisted reproduction and the investigation of aging and diseases related to SIRT1.

## Data availability statement

The original contributions presented in the study are included in the article/Supplementary material, further inquiries can be directed to the corresponding authors.

## Ethics statement

The management of ICR mice was approved by the Animal Care and Use Committee of South China Agricultural University (Approval number: 2022f116) during the entire experimental procedure.

## Author contributions

XX and ZL designed the research. XX, JP, JZ, and RS conducted the experiments. ZZ and ZHW performed the data analysis. XX, ZFW, and ZL wrote and edited the manuscript. ZZ and CW observed the study and joined in project administration. All authors contributed to the article and approved the submitted version.

## Funding

The research was funded by the Department of Science and Technology of Guangdong Province, China, grant numbers: 2022B0202090003 and 2019BT02N630 and the research was also supported by the Guangdong Modern Agricultural Industrial Technology System pig innovation team project, grant numbers: 2022KJ126.

## Conflict of interest

The authors declare that the research was conducted in the absence of any commercial or financial relationships that could be construed as a potential conflict of interest.

## Publisher’s note

All claims expressed in this article are solely those of the authors and do not necessarily represent those of their affiliated organizations, or those of the publisher, the editors and the reviewers. Any product that may be evaluated in this article, or claim that may be made by its manufacturer, is not guaranteed or endorsed by the publisher.

## Supplementary material

The Supplementary material for this article can be found online at: https://www.frontiersin.org/articles/10.3389/fnut.2023.1192758/full#supplementary-material

## References

[1] Hu LL Li HG Liao BY Xu Y Sun SC Wang JL. Exposure to nonylphenol impairs oocyte quality via the induction of organelle defects in mice. Ecotoxicol Environ Saf. (2022) 230:113136. doi: 10.1016/j.ecoenv.2021.113136 34995913

[2] Prasad S Tiwari M Pandey AN Shrivastav TG Chaube SK. Impact of stress on oocyte quality and reproductive outcome. J Biomed Sci. (2016) 23:36. doi: 10.1186/s12929-016-0253-4, PMID: 27026099 PMC4812655

[3] Zhang T Zhou Y Li L Wang HH Ma XS Qian WP. SIRT1, 2, 3 protect mouse oocytes from postovulatory aging. Aging. (2016) 8:685–94. doi: 10.18632/aging.100911, PMID: 26974211 PMC4925822

[4] Chen C Kattera S. Rescue ICSI of oocytes that failed to extrude the second polar body 6 h post-insemination in conventional IVF. Hum Reprod. (2003) 18:2118–21. doi: 10.1093/humrep/deg325, PMID: 14507831

[5] Xing X Zhang J Zhang J Wang Y Wang J Kang J. Coenzyme Q10 supplement rescues postovulatory oocyte aging by regulating SIRT4 expression. Curr Mol Pharmacol. (2022) 15:190–203. doi: 10.2174/1874467214666210420112819 33881976

[6] Tarin JJ Perez-Albala S Aguilar A Minarro J Hermenegildo C Cano A. Long-term effects of postovulatory aging of mouse oocytes on offspring: a two-generational study. Biol Reprod. (1999) 61:1347–55. doi: 10.1095/biolreprod61.5.1347, PMID: 10529284

[7] Tarin JJ Perez-Albala S Perez-Hoyos S Cano A. Postovulatory aging of oocytes decreases reproductive fitness and longevity of offspring. Biol Reprod. (2002) 66:495–9. doi: 10.1095/biolreprod66.2.495 11804967

[8] Van Blerkom J. Mitochondrial function in the human oocyte and embryo and their role in developmental competence. Mitochondrion. (2011) 11:797–813. doi: 10.1016/j.mito.2010.09.012 20933103

[9] Babayev E Seli E. Oocyte mitochondrial function and reproduction. Curr Opin Obstet Gynecol. (2015) 27:175–81. doi: 10.1097/GCO.0000000000000164 25719756 PMC4590773

[10] Yang Q Dai S Luo X Zhu J Li F Liu J. Melatonin attenuates postovulatory oocyte dysfunction by regulating SIRT1 expression. Reproduction. (2018) 156:81–92. doi: 10.1530/REP-18-0211 29752296

[11] Lin T Lee JE Kang JW Shin HY Lee JB Jin DI. Endoplasmic reticulum (ER) stress and unfolded protein response (UPR) in mammalian oocyte maturation and Preimplantation embryo development. Int J Mol Sci. (2019) 20:409. doi: 10.3390/ijms20020409 30669355 PMC6359168

[12] Park HJ Park JY Kim JW Yang SG Jung JM Kim MJ. Melatonin improves the meiotic maturation of porcine oocytes by reducing endoplasmic reticulum stress during in vitro maturation. J Pineal Res. (2018) 64:e12458. doi: 10.1111/jpi.12458, PMID: 29149522 PMC5814851

[13] Zhang JY Diao YF Kim HR Jin DI. Inhibition of endoplasmic reticulum stress improves mouse embryo development. PLoS One. (2012) 7:e40433. doi: 10.1371/journal.pone.0040433, PMID: 22808162 PMC3396646

[14] Takehara I Igarashi H Kawagoe J Matsuo K Takahashi K Nishi M. Impact of endoplasmic reticulum stress on oocyte aging mechanisms. Mol Hum Reprod. (2020) 26:567–75. doi: 10.1093/molehr/gaaa040, PMID: 32514562

[15] Moreno RD Schatten G Ramalho-Santos J. Golgi apparatus dynamics during mouse oocyte in vitro maturation: effect of the membrane trafficking inhibitor brefeldin a. Biol Reprod. (2002) 66:1259–66. doi: 10.1095/biolreprod66.5.1259, PMID: 11967185

[16] Racedo SE Rawe VY Niemann H. Dynamic changes of the Golgi apparatus during bovine in vitro oocyte maturation. Reproduction. (2012) 143:439–47. doi: 10.1530/REP-11-0492, PMID: 22301886

[17] Tsukamoto S Hara T Yamamoto A Ohta Y Wada A Ishida Y. Functional analysis of lysosomes during mouse preimplantation embryo development. J Reprod Dev. (2013) 59:33–9. doi: 10.1262/jrd.2012-096, PMID: 23080372 PMC3943237

[18] Miao JK Liu YH Liu S Liu XM Wang PC Du ZQ. Lysosomal dysfunction disturbs porcine oocyte maturation and developmental capacity by disorganizing chromosome/cytoskeleton and activating autophagy/apoptosis. Theriogenology. (2019) 140:44–51. doi: 10.1016/j.theriogenology.2019.08.019, PMID: 31437668

[19] McGinnis LK Pelech S Kinsey WH. Post-ovulatory aging of oocytes disrupts kinase signaling pathways and lysosome biogenesis. Mol Reprod Dev. (2014) 81:928–45. doi: 10.1002/mrd.22413, PMID: 25242074 PMC4211271

[20] Jia BY Xiang DC Shao QY Zhang B Liu SN Hong QH. Inhibitory effects of astaxanthin on postovulatory porcine oocyte aging in vitro. Sci Rep. (2020) 10:20217. doi: 10.1038/s41598-020-77359-6, PMID: 33214659 PMC7677382

[21] Chen C Zhou M Ge Y Wang X. SIRT1 and aging related signaling pathways. Mech Ageing Dev. (2020) 187:111215. doi: 10.1016/j.mad.2020.111215 32084459

[22] Tang BL. Sirt1 and the mitochondria. Mol Cells. (2016) 39:87–95. doi: 10.14348/molcells.2016.2318 26831453 PMC4757807

[23] Chandrasekaran K Anjaneyulu M Choi J Kumar P Salimian M Ho CY. Role of mitochondria in diabetic peripheral neuropathy: influencing the NAD(+)-dependent SIRT1-PGC-1alpha-TFAM pathway. Int Rev Neurobiol. (2019) 145:177–209. doi: 10.1016/bs.irn.2019.04.002, PMID: 31208524 PMC6590704

[24] Prola A Pires Da Silva J Guilbert A Lecru L Piquereau J Ribeiro M. SIRT1 protects the heart from ER stress-induced cell death through eIF2alpha deacetylation. Cell Death Differ. (2017) 24:343–56. doi: 10.1038/cdd.2016.138, PMID: 27911441 PMC5299716

[25] Xing X Zhang J Wu T Zhang J Wang Y Su J. SIRT1 reduces epigenetic and non-epigenetic changes to maintain the quality of postovulatory aged oocytes in mice. Exp Cell Res. (2021) 399:112421. doi: 10.1016/j.yexcr.2020.112421 33412164

[26] Pandurangan AK Esa NM. Luteolin, a bioflavonoid inhibits colorectal cancer through modulation of multiple signaling pathways: a review. Asian Pac J Cancer Prev. (2014) 15:5501–8. doi: 10.7314/APJCP.2014.15.14.5501, PMID: 25081655

[27] Imran M Rauf A Abu-Izneid T Nadeem M Shariati MA Khan IA. Luteolin, a flavonoid, as an anticancer agent: a review. Biomed Pharmacother. (2019) 112:108612. doi: 10.1016/j.biopha.2019.108612 30798142

[28] Kim A Lee W Yun JM. Luteolin and fisetin suppress oxidative stress by modulating sirtuins and forkhead box O3a expression under in vitro diabetic conditions. Nutr Res Pract. (2017) 11:430–4. doi: 10.4162/nrp.2017.11.5.430, PMID: 28989580 PMC5621366

[29] Le Marchand L. Cancer preventive effects of flavonoids--a review. Biomed Pharmacother. (2002) 56:296–301. doi: 10.1016/S0753-3322(02)00186-5, PMID: 12224601

[30] Aziz N Kim MY Cho JY. Anti-inflammatory effects of luteolin: a review of in vitro, in vivo, and in silico studies. J Ethnopharmacol. (2018) 225:342–58. doi: 10.1016/j.jep.2018.05.019, PMID: 29801717

[31] Sun MH Li XH Xu Y Xu Y Pan ZN Sun SC. Citrinin exposure disrupts organelle distribution and functions in mouse oocytes. Environ Res. (2020) 185:109476. doi: 10.1016/j.envres.2020.109476, PMID: 32278162

[32] Zhu RZ Li BS Gao SS Seo JH Choi BM. Luteolin inhibits H2O2-induced cellular senescence via modulation of SIRT1 and p53. Korean J Physiol Pharmacol. (2021) 25:297–305. doi: 10.4196/kjpp.2021.25.4.297, PMID: 34187948 PMC8255127

[33] Wang L Tang J Wang L Tan F Song H Zhou J. Oxidative stress in oocyte aging and female reproduction. J Cell Physiol. (2021) 236:7966–83. doi: 10.1002/jcp.30468 34121193

[34] Lee AR Thanh Ha L Kishigami S Hosoi Y. Abnormal lysine acetylation with postovulatory oocyte aging. Reprod Med Biol. (2014) 13:81–6. doi: 10.1007/s12522-013-0172-y, PMID: 29699152 PMC5906867

[35] Hisahara S Iwahara N Matsushita T Suzuki S Matsumura A Fujikura M. SIRT1 decelerates morphological processing of oligodendrocyte cell lines and regulates the expression of cytoskeleton-related oligodendrocyte proteins. Biochem Biophys Res Commun. (2021) 546:7–14. doi: 10.1016/j.bbrc.2021.01.095, PMID: 33556638

[36] Dumollard R Duchen M Carroll J. The role of mitochondrial function in the oocyte and embryo. Curr Top Dev Biol. (2007) 77:21–49. doi: 10.1016/S0070-2153(06)77002-8 17222699

[37] Yadav RK Chae SW Kim HR Chae HJ. Endoplasmic reticulum stress and cancer. J Cancer Prev. (2014) 19:75–88. doi: 10.15430/JCP.2014.19.2.75, PMID: 25337575 PMC4204165

[38] Gao J Gao A Liu W Chen L. Golgi stress response: a regulatory mechanism of Golgi function. Biofactors. (2021) 47:964–74. doi: 10.1002/biof.1780 34500494

[39] Wang H Zhong C Yang R Yin Y Tan R Gao L. Hfm1 participates in Golgi-associated spindle assembly and division in mouse oocyte meiosis. Cell Death Dis. (2020) 11:490. doi: 10.1038/s41419-020-2697-4 32606310 PMC7327073

[40] Ng MM Dippold HC Buschman MD Noakes CJ Field SJ. GOLPH3L antagonizes GOLPH3 to determine Golgi morphology. Mol Biol Cell. (2013) 24:796–808. doi: 10.1091/mbc.e12-07-0525, PMID: 23345592 PMC3596250

[41] Bonam SR Wang F Muller S. Lysosomes as a therapeutic target. Nat Rev Drug Discov. (2019) 18:923–48. doi: 10.1038/s41573-019-0036-1, PMID: 31477883 PMC7097195

[42] Sun YL Tang SB Shen W Yin S Sun QY. Roles of resveratrol in improving the quality of postovulatory aging oocytes in vitro. Cells. (2019) 8:1132. doi: 10.3390/cells8101132, PMID: 31547622 PMC6829324

[43] Wang H Jo YJ Oh JS Kim NH. Quercetin delays postovulatory aging of mouse oocytes by regulating SIRT expression and MPF activity. Oncotarget. (2017) 8:38631–41. doi: 10.18632/oncotarget.16219 28418847 PMC5503559

[44] Yang DQ Tan X Lv ZJ Liu BY Baiyun RQ Lu JJ. Regulation of Sirt1/Nrf2/TNF-alpha signaling pathway by luteolin is critical to attenuate acute mercuric chloride exposure induced hepatotoxicity. Sci Rep. (2016) 6:37157. doi: 10.1038/srep37157 27853236 PMC5112569

[45] Wang Q Wang HD Jia Y Pan H Ding H. Luteolin induces apoptosis by ROS/ER stress and mitochondrial dysfunction in gliomablastoma. Cancer Chemother Pharmacol. (2017) 79:1031–41. doi: 10.1007/s00280-017-3299-4, PMID: 28393257

[46] Hytti M Szabo D Piippo N Korhonen E Honkakoski P Kaarniranta K. Two dietary polyphenols, fisetin and luteolin, reduce inflammation but augment DNA damage-induced toxicity in human RPE cells. J Nutr Biochem. (2017) 42:37–42. doi: 10.1016/j.jnutbio.2016.12.014, PMID: 28113103

[47] Rahman S Islam R. Mammalian Sirt1: insights on its biological functions. Cell Commun Signal. (2011) 9:11. doi: 10.1186/1478-811X-9-11, PMID: 21549004 PMC3103488

[48] Lin Z Fang D. The roles of SIRT1 in cancer. Genes Cancer. (2013) 4:97–104. doi: 10.1177/1947601912475079, PMID: 24020000 PMC3764469

[49] Chen GD Yu WD Chen XP. SirT1 activator represses the transcription of TNFalpha in THP1 cells of a sepsis model via deacetylation of H4K16. Mol Med Rep. (2016) 14:5544–50. doi: 10.3892/mmr.2016.5942, PMID: 27878240 PMC5355689

[50] Rifai K Judes G Idrissou M Daures M Bignon YJ Penault-Llorca F. SIRT1-dependent epigenetic regulation of H3 and H4 histone acetylation in human breast cancer. Oncotarget. (2018) 9:30661–78. doi: 10.18632/oncotarget.25771, PMID: 30093977 PMC6078139

[51] Huang J Zhang F Hu G Pan Y Sun W Jiang L. SIRT1 suppresses pituitary tumor progression by downregulating PTTG1 expression. Oncol Rep. (2022) 48:143. doi: 10.3892/or.2022.8354, PMID: 35730625

[52] Di Emidio G Falone S Vitti M D'Alessandro AM Vento M Di Pietro C. SIRT1 signalling protects mouse oocytes against oxidative stress and is deregulated during aging. Hum Reprod. (2014) 29:2006–17. doi: 10.1093/humrep/deu160 24963165

[53] van der Horst A Burgering BM. Stressing the role of FoxO proteins in lifespan and disease. Nat Rev Mol Cell Biol. (2007) 8:440–50. doi: 10.1038/nrm2190 17522590

[54] Wang F Yao S Xia H. SIRT1 is a key regulatory target for the treatment of the endoplasmic reticulum stress-related organ damage. Biomed Pharmacother. (2020) 130:110601. doi: 10.1016/j.biopha.2020.110601, PMID: 32784049

[55] Zhang K Zhang M Zhao H Yan B Zhang D Liang J. S100A4 regulates motility and invasiveness of human esophageal squamous cell carcinoma through modulating the AKT/slug signal pathway. Dis Esophagus. (2012) 25:731–9. doi: 10.1111/j.1442-2050.2012.01323.x 22458600

[56] Schwarz DS Blower MD. The endoplasmic reticulum: structure, function and response to cellular signaling. Cell Mol Life Sci. (2016) 73:79–94. doi: 10.1007/s00018-015-2052-6, PMID: 26433683 PMC4700099

[57] Pan ZN Pan MH Sun MH Li XH Zhang Y Sun SC. RAB7 GTPase regulates actin dynamics for DRP1-mediated mitochondria function and spindle migration in mouse oocyte meiosis. FASEB J. (2020) 34:9615–27. doi: 10.1096/fj.201903013R, PMID: 32472654

[58] Sun MH Hu LL Zhao CY Lu X Ren YP Wang JL. Ral GTPase is essential for actin dynamics and Golgi apparatus distribution in mouse oocyte maturation. Cell Div. (2021) 16:3. doi: 10.1186/s13008-021-00071-y, PMID: 34112192 PMC8194175

[59] Ma R Zhang J Liu X Li L Liu H Rui R. Involvement of Rab6a in organelle rearrangement and cytoskeletal organization during mouse oocyte maturation. Sci Rep. (2016) 6:23560. doi: 10.1038/srep23560, PMID: 27030207 PMC4814827

[60] Kim B Zhang X Kan R Cohen R Mukai C Travis AJ. The role of MATER in endoplasmic reticulum distribution and calcium homeostasis in mouse oocytes. Dev Biol. (2014) 386:331–9. doi: 10.1016/j.ydbio.2013.12.025, PMID: 24374158 PMC3960596

[61] Lu X Gao Z Qin D Li L. A maternal functional module in the mammalian oocyte-to-embryo transition. Trends Mol Med. (2017) 23:1014–23. doi: 10.1016/j.molmed.2017.09.004, PMID: 28993030

[62] Wang Y Xu Y Ju JQ Liu JC Sun SC. Fumonisin B1 exposure deteriorates oocyte quality by inducing organelle dysfunction and DNA damage in mice. Ecotoxicol Environ Saf. (2021) 223:112598. doi: 10.1016/j.ecoenv.2021.112598 34388657

[63] Wang Y Pan ZN Xing CH Zhang HL Sun SC. Nivalenol affects spindle formation and organelle functions during mouse oocyte maturation. Toxicol Appl Pharmacol. (2022) 436:115882. doi: 10.1016/j.taap.2022.115882, PMID: 35016910

[64] Zhang YZ Zhao QH Duan HW Zou YJ Sun SC Hu LL. Aflatoxin B1 exposure disrupts organelle distribution in mouse oocytes. PeerJ. (2022) 10:e13497. doi: 10.7717/peerj.13497, PMID: 35646486 PMC9135037

[65] Pan MH Wu YK Liao BY Zhang H Li C Wang JL. Bisphenol a exposure disrupts organelle distribution and functions during mouse oocyte maturation. Front Cell Dev Biol. (2021) 9:661155. doi: 10.3389/fcell.2021.661155, PMID: 33834027 PMC8021768

[66] Coticchio G Lagalla C Sturmey R Pennetta F Borini A. The enigmatic morula: mechanisms of development, cell fate determination, self-correction and implications for ART. Hum Reprod Update. (2019) 25:422–38. doi: 10.1093/humupd/dmz008, PMID: 30855681

