# Supplementary material for: Luteolin regulates the distribution and function of organelles by controlling SIRT1 activity during postovulatory oocyte aging
Source: Front Nutr. 2023 Jul 31;10:1192758. doi: 10.3389/fnut.2023.1192758 (PMC10424794; doi:10.3389/fnut.2023.1192758)
Supplement: Supplementary file 1 [file Data_Sheet_1.docx]

**Supplement table 1**

Primer sequences for real time PCR

| Gene | Sequence |
| --- | --- |
| *Gapdh* | (F) 5’- TCTTGCTCAGTGTCCTTGC-3’ (R) 5’- CTTTGTCAAGCTCATTTCCTGG-3’ |
| *Sirt1* | (F) 5’- CTCTGAAAGTGAGACCAGTAGC-3’ (R) 5’- TGTAGATGAGGCAAAGGTTCC-3’ |


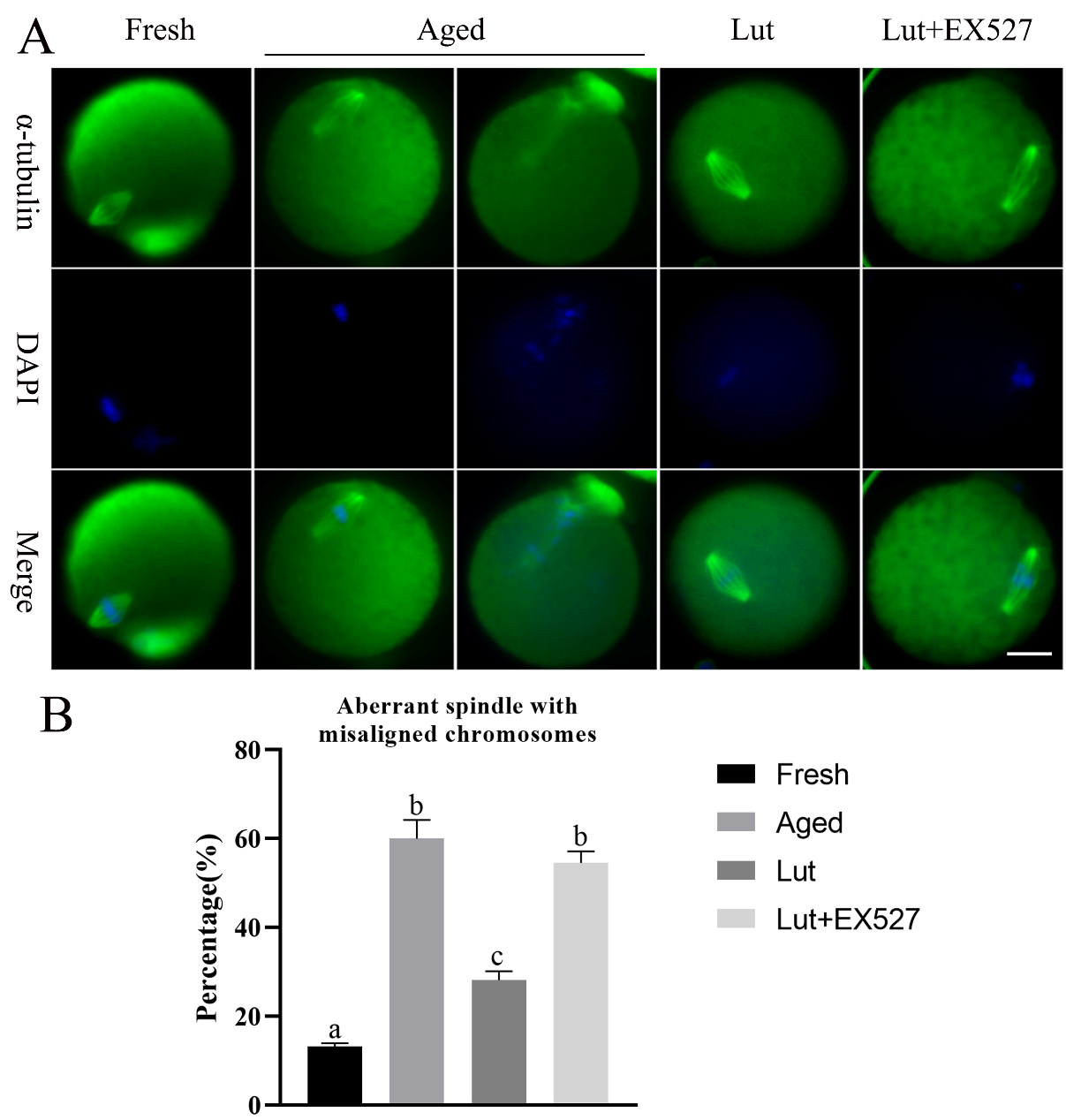


Figure S1. Effects of luteolin on spindle morphologies. (A) The representative figures of α-tubulin and DNA in Fresh, aged, Lut, and Lut+EX527 oocytes. Scale bar, 30 μm. (B) Analysis the proportion of abnormal spindles with misaligned chromosomes from different groups (n = 60 for each group). Different superscripts of a b c mean differences at P < 0.05.

**Table S2.** The rate of 2-cell and blastocyst formation after fertilization of oocytes derived from fresh groups and different dose of Luteolin treatment for 12 h.

| Lut (uM) | n (total embryos) | n (2-cell) (%, mean ± SEM) | n (blastocyst) (%, mean ± SEM) |
| --- | --- | --- | --- |
| Fresh | 120 | 98 (81.80 ± 3.25) ^a^ | 89 (74.05 ± 1.46) ^a^ |
| 0 | 120 | 82 (64.17 ± 3.53) ^b^ | 42 (35.00 ± 3.84) ^b^ |
| 1 | 116 | 80 (68.97 ± 1.73) ^b^ | 54 (46.55 ± 2.93) ^c^ |
| 3 | 108 | 80 (74.07 ± 2.66) ^b^ | 60 (55.56 ± 3.44) ^dc^ |
| 5 | 125 | 95 (76.00 ± 3.85) ^c^ | 76 (60.80 ± 3.58) ^d^ |
| 10 | 112 | 74 (66.07 ± 2.31) ^b^ | 57 (50.89 ± 2,56) ^c^ |

**Table S3**. The rate of 2-cell and blastocyst formation after fertilization of oocytes derived from aged groups and different dose of EX527 treatment for 12 h. data shown as mean ± SEM.

| Aged+EX527(μM) | n (total embryos) | 24 h | d 5 | |
| --- | --- | --- | --- | --- |
|  |  | n (2-cell) (%) | | n (blastocyst) (%) |
| 0 | 114 | 67 (57.74 ± 6.40) ^a^ | | 46 (39.69 ± 3.53) ^a^ |
| 0.2 | 112 | 60 (54.74 ± 3.80) ^a^ | | 41 (35.33 ± 3.82) ^a^ |
| 0.5 | 99 | 62 (62.70 ± 1.91) ^a^ | | 32 (32.16 ± 2.99) ^a^ |
| 1 | 109 | 75 (68.24 ± 5.60) ^a^ | | 40 (35.73 ± 9.30) ^a^ |

**Table S4.** The formation proportion of 2-cell, morula, and blastocyst after fertilization of oocytes derived from 5 μM Luteolin combined with different dose of EX527 treatment for 12 h. data shown as mean ± SEM.

| Lut (μM) + EX527(μM) | n (total embryos) | 24 h | d 4.5 | | d 5.5 |
| --- | --- | --- | --- | --- | --- |
|  |  | n (2-cell) (%) | n (morula) (%) | n (blastocyst) (%) | n (blastocyst) (%) |
| 0 + 0 | 120 | 82 (68.3 ± 2.98 )^a^ | 34 (28.33± 2.47) ^a^ | 10 (8.33 ± 2.83) ^a^ | 43 (35.83 ± 4.34)^a^ |
| 5 + 0 | 110 | 84 (76.36 ± 3.17)^b^ | 4 (3.63 ± 0.94) ^b^ | 68 (61.82 ± 2.89)^b^ | 70 (63.64 ± 2.04) ^b^ |
| 5 + 0.1 | 109 | 81 (74.31± 2.14) ^b^ | 17 (15.60 ± 1.33) ^c^ | 40 (36.70 ± 5.77) ^c^ | 56 (51.38 ± 5.04) ^c^ |
| 5 + 0.2 | 121 | 89 (73.55 ± 3.04) ^ab^ | 38 (31.40 ± 3.20) ^a^ | 28 (23.14 ± 2.35) ^d^ | 63 (52.07 ± 2.01) ^c^ |
| 5 + 0.5 | 123 | 92 (74.80 ± 2.45) ^b^ | 43 (34.96 ± 1.33) ^a^ | 22 (17.89 ± 1.77) ^d^ | 62 (50.41 ± 3.12) ^c^ |
| 5 + 1 | 102 | 77 (75.49 ± 6.04) ^b^ | 20 (19.61 ± 3.45) ^c^ | 33 (32.35 ± 2.17) ^c^ | 53 (51.96 ± 3.46) ^c^ |


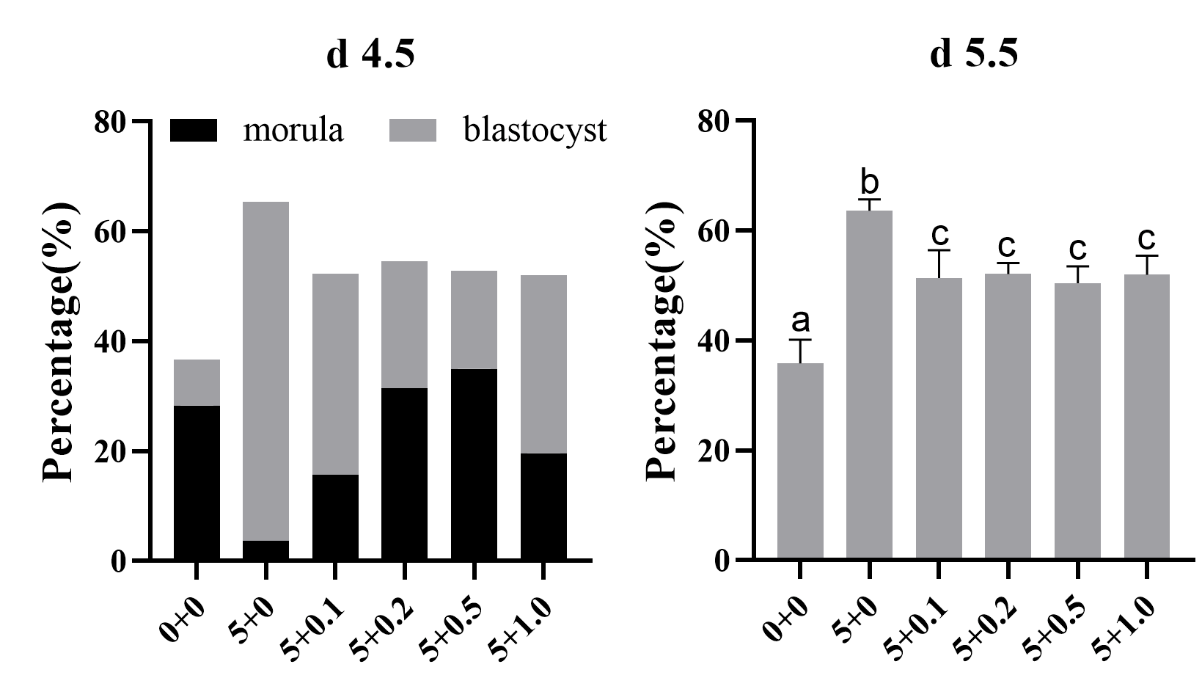


Figure S2. Effects of EX527 on the development of embryos. (A) The percentage of morula and blastocyst at day 4.5 derived from aged groups, and different co-treatment groups of 5 μM luteolin with varying concentrations of EX527 (X: luteolin (μM) + EX527 (μM)). (B) The percentage of blastocyst at day 5.5 derived from aged groups, and different co-treatment groups of 5 μM luteolin with varying concentrations of EX527 (X: luteolin (μM) + EX527 (μM)). Different superscripts of a b c mean differences at P < 0.05.


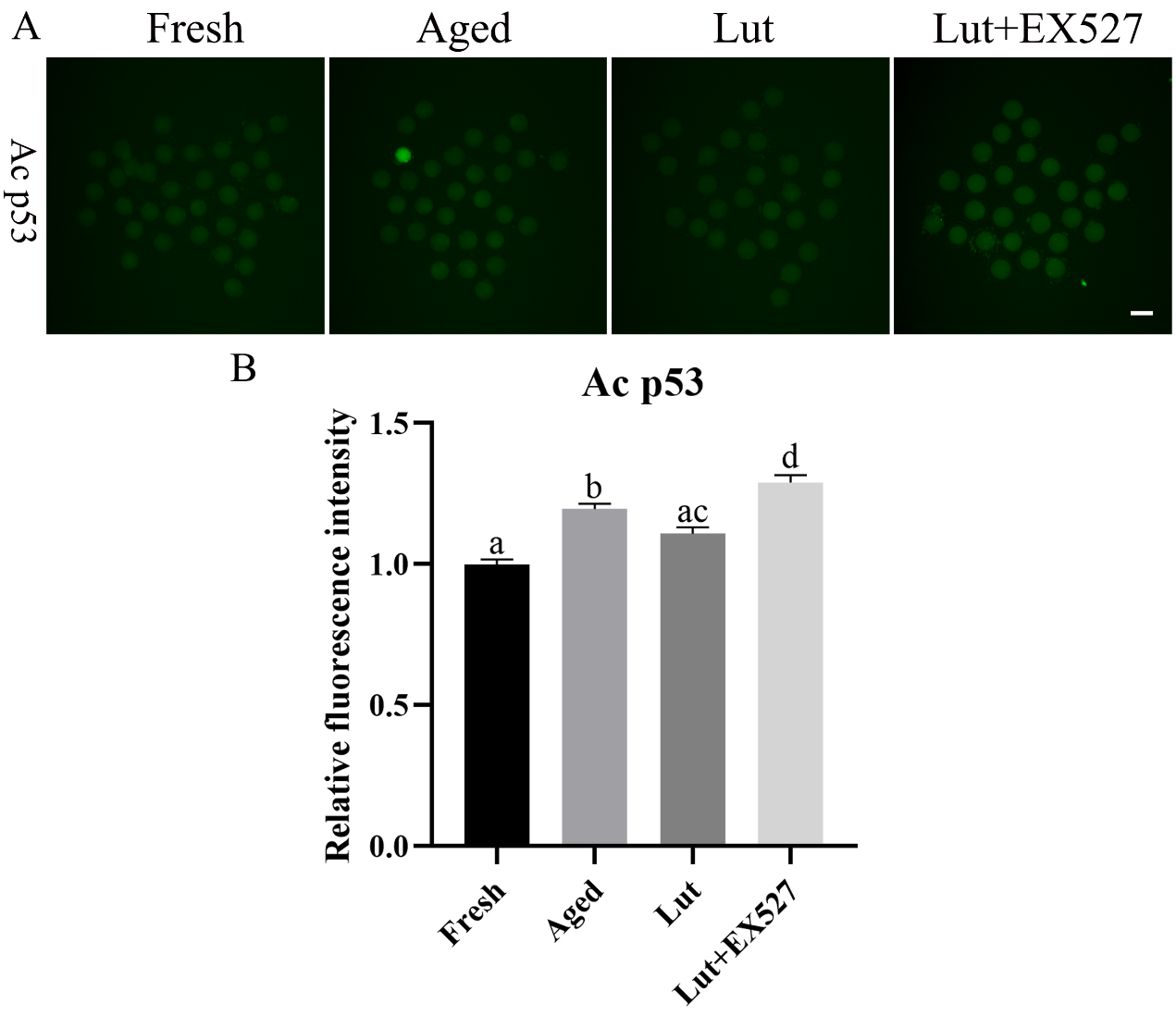


Figure S3. The impact of luteolin on acetylation of P53. (A) The representative pictures of ac p53 in oocytes derived from Fresh, Aged, Lut, and Lut+EX527 groups. Scale bar, 60 μm. 5s, 5.1x. (B) Analysis of relative intensity of ac p53 signals in oocytes from different groups (n = 20 for each group). Difference of superscripts indicates p < 0.05.
